# Avoiding the Enumeration of Infeasible Elementary Flux Modes by Including Transcriptional Regulatory Rules in the Enumeration Process Saves Computational Costs

**DOI:** 10.1371/journal.pone.0129840

**Published:** 2015-06-19

**Authors:** Christian Jungreuthmayer, David E. Ruckerbauer, Matthias P. Gerstl, Michael Hanscho, Jürgen Zanghellini

**Affiliations:** 1 Austrian Centre of Industrial Biotechnology, Vienna, Austria, EU; 2 Department of Biotechnology, University of Natural Resources and Life Sciences, Vienna, Austria, EU; Université de Nantes, FRANCE

## Abstract

Despite the significant progress made in recent years, the computation of the complete set of elementary flux modes of large or even genome-scale metabolic networks is still impossible. We introduce a novel approach to speed up the calculation of elementary flux modes by including transcriptional regulatory information into the analysis of metabolic networks. Taking into account gene regulation dramatically reduces the solution space and allows the presented algorithm to constantly eliminate biologically infeasible modes at an early stage of the computation procedure. Thereby, computational costs, such as runtime, memory usage, and disk space, are extremely reduced. Moreover, we show that the application of transcriptional rules identifies non-trivial system-wide effects on metabolism. Using the presented algorithm pushes the size of metabolic networks that can be studied by elementary flux modes to new and much higher limits without the loss of predictive quality. This makes unbiased, system-wide predictions in large scale metabolic networks possible without resorting to any optimization principle.

## Introduction

Elementary flux modes (EFMs) are indivisible sets of reactions that represent biologically meaningful pathways [1, 2] under steady state condition. Removing only a single reaction of an EFM results in the extinction of the entire pathway. EFMs can be used to mathematically decompose metabolic networks into minimal functional building blocks and investigate them unbiasedly. For that reason EFMs have gained increasing attention in the field of metabolic engineering in recent years [3]. However, the computational costs for calculating EFMs increase sharply with the size of the analyzed network [4]. The calculation of all EFMs of small networks (up to 50 reactions) is straightforward and simple. Despite the major progress made recently [5–8] the computation of the complete set of EFMs of large scale networks is still very challenging if not impossible. There is a number of tools specifically designed to calculate the complete set of EFMs as efficiently as possible, such as *Metatool*[9], *CellNetAnalyzer*[10] and *efmtool*[6]. The *efmtool* written by Marco Terzer is—to the best of our knowledge—currently the fastest program available [11]. It is written in the multi-platform programming language *Java*, supports multi-threading, is published under the open source software license *Simplified BSD Style License*[12], and can be downloaded from [13].

In the presented work we introduce a novel approach to speed up the computation of the complete set of biologically feasible EFMs. Our aim is to not enumerate all topologically feasible EFMs, but only the much smaller subset of biologically relevant EFMs. To this end we take into account the gene regulatory information of the investigated metabolic network.

Basically, there are two main ways to model gene regulatory networks: a) discrete models, such as Boolean models and Petri networks and b) continuous models, such as linear models and differential equation models [14]. The determination of the parameters of continuous models is non-trivial [15]. Hence, transcriptional regulatory networks (TRN) are often provided as a Boolean rule set, e.g. [14–18]. These rules exclude many of the topologically feasible EFMs for biological reasons. We implemented our algorithm by extending *efmtool*, thereby, exploiting the full power and advantage of open source software. By utilizing a specific feature of the binary approach [5] which was applied in *efmtool*, the elimination of biologically infeasible modes can be done constantly and at an early stage of the EFM computation process. Thereby, a huge reduction of the computational costs, such as execution time, memory consumption and hard disk space, is achieved.

## Methods

### Binary approach

Modern EFM computation programs, such as *efmtool*, use a binary approach [5] of the double description method (DDM) [19]. In the following we briefly review this binary approach. We will introduce our modifications for the inclusion of transcriptional regulation in the next section.

The binary approach is characterized by splitting each mode into a binary part and a numerical part. The binary part of a mode contains only a single bit for each reaction, where ‘1’ means that the reaction carries a flux and ‘0’ stands for a reaction not carrying a flux. While iterating through the algorithm, the numerical part of each mode is successively converted into the binary representation. The iteration procedure terminates when each mode has been completely transformed to its binary form. In a final post-iteration step the computed binary modes are converted back to their numerical forms. The numerical representation of a mode gives the exact stoichiometric flux values of each participating reaction.

**Fig 1:**
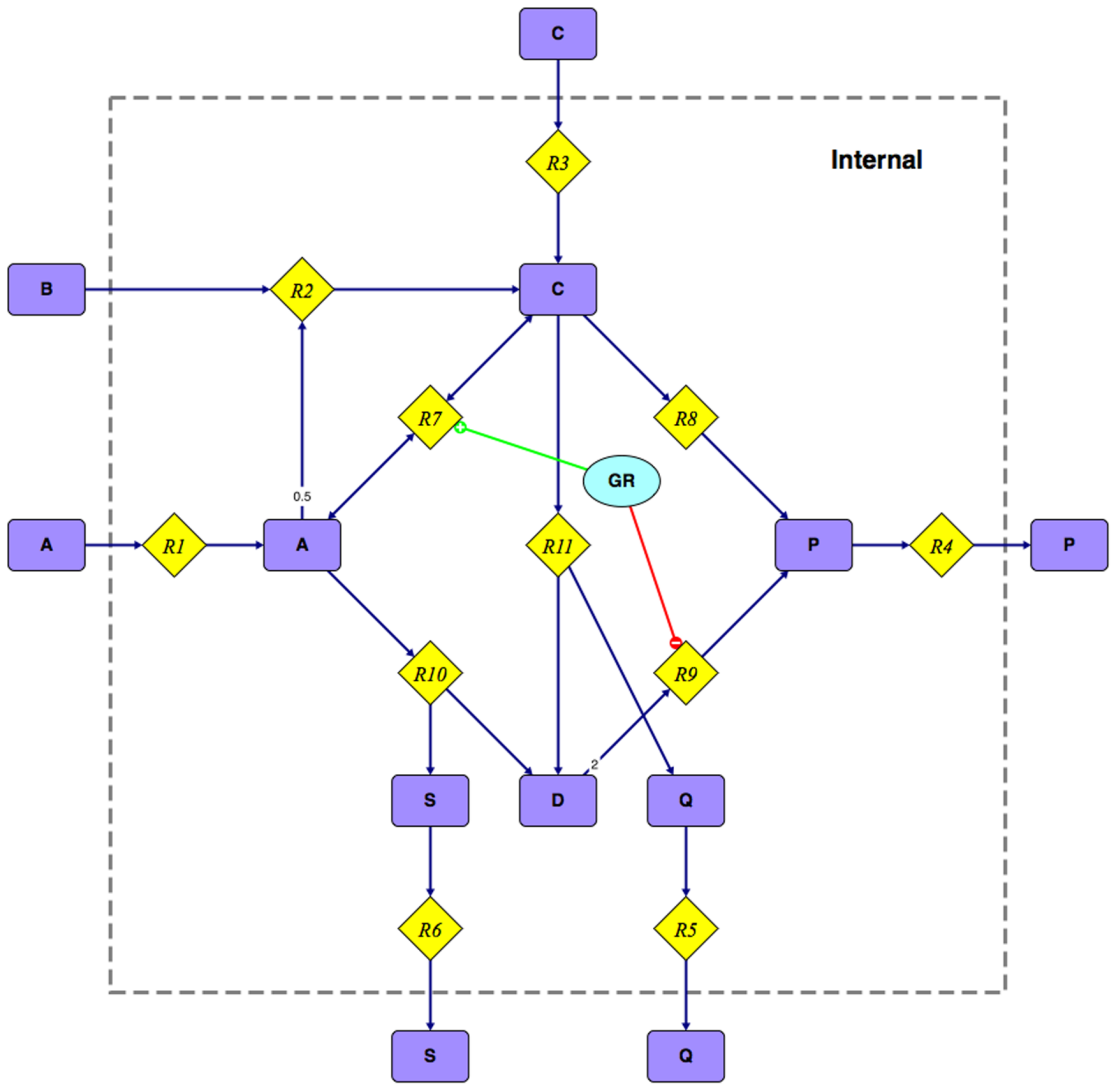
Example network consisting of 12 metabolites (rectangles), 11 reactions (diamonds), and a gene regulatory network: *R*7*r* = NOT(*R*9). All reactions are irreversible, except for R7r.

We demonstrate the general principles of the binary approach with the simple example network shown in Fig 1. For the sake of clarity the network will not be compressed in order to keep all originally specified reactions and metabolites of the network. In a ‘real-life’ computation several compression strategies would be applied first in order to combine and remove topographically redundant reactions and metabolites [5, 20]. The internal stoichiometric matrix, *S*, of the example network is shown in S1 Table. External metabolites act as sources and sinks for EFMs, they do not obey the steady state condition and are therefore omitted in *S*.

First, the reversible reaction R7r is split into a forward and a backward irreversible reaction. This is done by negating the column of the reversible reaction and appending the newly created column right after the original one. S2 Table shows the extended stoichiometric matrix, *S*_ext_, that only contains irreversible reactions.

The main process of computing all EFMs is based on the DDM [19]. First DDM determines an initial set of intermediate EFMs which are then iteratively combined and added to the set of existing EFMs until the complete set of final EFMs is obtained. The (intermediate) EFMs are stored in the mode matrix, *R*, that contains one column for each EFM. Typically, the initialization of the mode matrix, *R*, is obtained by calculating the kernel, *K*, of the extended stoichiometric matrix, *S*_ext_. *K* is defined by *S*_ext_ *K* = 0 and is shown in Table 1.

**Table 1:** Kernel matrix *K* of the extended stoichiometric matrix shown in S2 Table.

R1	-0.5	0.5	-0.5	0.5	1.0	0.5
R2	-1.0	-1.0	1.0	1.0	0.0	1.0
R3	1.0	0.0	0.0	0.0	0.0	0.0
R4	0.0	0.0	0.0	1.0	0.5	0.5
R5	0.0	0.0	0.0	0.0	0.0	1.0
R6	0.0	0.0	0.0	0.0	1.0	0.0
R7f	0.0	1.0	0.0	0.0	0.0	0.0
R7b	0.0	0.0	1.0	0.0	0.0	0.0
R8	0.0	0.0	0.0	1.0	0.0	0.0
R9	0.0	0.0	0.0	0.0	0.5	0.5
R10	0.0	0.0	0.0	0.0	1.0	0.0
R11	0.0	0.0	0.0	0.0	0.0	1.0

Next, the initial conversion to the binary representation of the mode matrix, *R*, is performed. The final set of EFMs of the extended network must only contain non-negative flux values, as the extended network contains only irreversible reactions. As pointed out by Gagneur and Klamt [5] using only irreversible reactions is of major importance, as all non-zero elements of a mode will be retained if a new mode is created by combining this mode with other modes that have already been determined during the calculation procedure. All rows that contain only non-negative values can directly be transformed to the binary representation. For the sake of clarity we use the character “t” for *true* or binary “1” indicating a flux carrying reaction and the character “f” for *false* or binary “0” indicating that no flux occurs. Usually, the initial mode matrix, *R*, is sorted in a way that all rows containing only positive values are at the top. Table 2 shows the properly sorted mode matrix, *R*, containing binary and numerical values before the iteration process is started. Note that R1 and R2 were re-ordered for computational reasons. The order of the reactions has no effect on the final set of computed EFMs, but might have a significant influence on the performance of the nullspace algorithm [21].

**Table 2:** Initial mode matrix *R* for EFM calculation. Note that the order of the reactions has changed to maximize the number of leading rows that can directly be converted to binary form in the pre-iteration phase. The first ten rows were already transformed to the binary representation. Here “t” represents a binary “1” (true) and indicates that the reaction carries flux. “f” stands for a binary “0” (false) and indicates that the reaction flux is zero.

	M1	M2	M3	M4	M5	M6
R3	t	f	f	f	f	f
R4	f	f	f	t	t	t
R5	f	f	f	f	f	t
R6	f	f	f	f	t	f
R7f	f	t	f	f	f	f
R7b	f	f	t	f	f	f
R8	f	f	f	t	f	f
R9	f	f	f	f	t	t
R10	f	f	f	f	t	f
R11	f	f	f	f	f	t
R2	-1.0	-1.0	1.0	1.0	0.0	1.0
R1	-0.5	0.5	-0.5	0.5	1.0	0.5

Next, the iteration procedure is performed. Step by step each row that is still in numerical form is transformed to its binary representation. As shown in Table 2 the next reaction to be processed is *R2*. The DDM requires that all modes containing non-negative values at *R2* are retained, whereas the modes with negative values are removed. Furthermore, the method requires that all modes with negative values at *R2* are combined with adjacent modes exhibiting a positive value at *R2*. Hence, the modes M1 and M2 are combined with M3, M4, and M6 resulting in six potential new modes. Two modes are adjacent if the binary part of the new mode is not a superset of any already existing modes—except the two parent modes. For the binary part, the combination of two adjacent modes is a simple and fast bitwise OR operation of the involved modes. Combining the numerical part is achieved by a weighted subtraction of the two numerical vectors. The new numerical value, *v*_new_*r*__, of row *r* is calculated by $v_{{\text{new}}_{r}}=(v_{1}^{+}v_{r}^{-}-v_{1}^{-}v_{r}^{+})/(v_{1}^{+}-v_{1}^{-})$, where $v_{r}^{+}$ and $v_{r}^{-}$ are the values of the positive (+) and of the negative (-) column at row *r*, respectively. The row index *r* runs from 1 to *n*, where row *r* = 1 is the row to be converted at current iteration step and *n* is the number of rows left to be converted. By construction, *v*_new_1__ = 0.0, and thus can directly be converted to binary form. All other values *v*_new_*r*__, *r* ∈ [2, *n*] will be converted in successive iteration steps. Applying these instructions to the initial mode matrix, *R*, given in Table 2 results in the new mode matrix shown in Table 3.

**Table 3:** Mode matrix *R* after the first iteration step (left) converting reaction *R2* from numerical to binary form and after the last iteration step (right) for an ordinary EFM analysis. After the final step *R* contains only binary values. In the final matrix M6 is a futile 2-cycle mode and can be removed.

	After first step										After last step											
	M1	M2	M3	M4	M5	M6	M7	M8	M9	M10	M1	M2	M3	M4	M5	M6	M7	M8	M9	M10	M11	M12
R3	f	f	f	f	f	f	f	t	t	t	f	f	f	f	f	f	t	t	t	f	f	f
R4	f	t	t	t	t	t	f	t	t	f	t	t	t	t	t	f	t	t	t	t	t	t
R5	f	f	f	t	t	f	f	t	f	f	f	f	t	t	f	f	t	f	f	t	f	f
R6	f	f	t	f	f	f	f	f	f	f	f	t	f	f	f	f	f	f	t	f	t	f
R7f	f	f	f	f	t	t	t	f	f	f	f	f	f	t	t	t	f	f	f	f	f	f
R7b	t	f	f	f	f	f	t	f	f	t	f	f	f	f	f	t	f	f	t	t	t	t
R8	f	t	f	f	f	t	f	f	t	f	t	f	f	f	t	f	f	t	f	f	f	t
R9	f	f	t	t	t	f	f	t	f	f	f	t	t	t	f	f	t	f	t	t	t	f
R10	f	f	t	f	f	f	f	f	f	f	f	t	f	f	f	f	f	f	t	f	t	f
R11	f	f	f	t	t	f	f	t	f	f	f	f	t	t	f	f	t	f	f	t	f	f
R2	t	t	f	t	f	f	f	f	f	f	t	f	t	f	f	f	f	f	f	t	t	t
R1	-0.5	0.5	1.0	0.5	0.5	0.5	0.0	0.0	0.0	-0.5	t	t	t	t	t	f	f	f	f	f	f	f

Applying the mode combination procedure again for the last row to be converted (*R1*) results in the final mode matrix, *R*, as shown in Table 3. Now, the matrix, *R*, contains only binary elements. Note that the performance of the described iteration procedure for ‘real-life’ networks can be tremendously increased if tree structures are utilized to store the binary representation of the modes [6].

Next, the futile 2-cycle mode (M6) that was created by splitting the reversible reaction *R7r* is removed. Then the irreversible forward and backward reactions *R7f* and *R7b* are combined by a simple bitwise OR operation in order to obtain the reversible reaction *R7r* again. The final set of modes in binary form is shown in S3 Table.

Recovering the numerical representation is achieved by using the fact that the reduced nullspace matrix, *N*_red_, multiplied by the sought numerical mode has dimension one and is equal to zero [5]. *N*_red_ is a sub-matrix of the kernel, *K*, that only contains columns/reactions where the binary mode to be transformed carries a flux. Hence, only a homogeneous linear system has to be solved to obtain the 1-dimensional solution vector that represents the numerical form of a mode. The result of this re-conversion for the simple example network is listed in Table 4.

**Table 4:** Numerical representation of all EFMs of the example network shown in Fig 1 calculated with ordinary EFMtool (left) and regEFMtool (right) using the gene regulatory rule *R*7*r* = NOT(*R*9). Note that the irreversible reactions R7f and Rfb were combined to the reversible reaction R7r and the futile two-cycle mode caused by R7r was removed.

	EFMtool											regEFMtool										
	R1	R2	R3	R4	R5	R6	R7r	R8	R9	R10	R11	R1	R2	R3	R4	R5	R6	R7r	R8	R9	R10	R11
EFM01	0.00	1.00	0.00	0.50	0.00	0.00	-0.50	0.50	0.00	0.00	0.00	0.50	1.00	0.00	0.50	1.00	0.00	0.00	0.00	0.50	0.00	1.00
EFM02	0.00	1.00	0.00	0.25	0.50	0.00	-0.50	0.00	0.25	0.00	0.50	0.00	1.00	0.00	0.50	0.00	0.00	-0.50	0.50	0.00	0.00	0.00
EFM03	0.00	1.00	0.00	0.25	0.00	0.50	-1.00	0.00	0.25	0.50	0.00	1.00	0.00	0.00	0.50	0.00	1.00	0.00	0.00	0.50	1.00	0.00
EFM04	0.00	0.00	1.00	0.50	0.00	1.00	-1.00	0.00	0.50	1.00	0.00	1.00	0.00	0.00	1.00	0.00	0.00	1.00	1.00	0.00	0.00	0.00
EFM05	0.50	1.00	0.00	1.00	0.00	0.00	0.00	1.00	0.00	0.00	0.00	0.00	0.00	1.00	0.50	1.00	0.00	0.00	0.00	0.50	0.00	1.00
EFM06	0.50	1.00	0.00	0.50	1.00	0.00	0.00	0.00	0.50	0.00	1.00											
EFM07	1.00	0.00	0.00	0.50	0.00	1.00	0.00	0.00	0.50	1.00	0.00											
EFM08	1.00	0.00	0.00	0.50	1.00	0.00	1.00	0.00	0.50	0.00	1.00											
EFM09	1.00	0.00	0.00	1.00	0.00	0.00	1.00	1.00	0.00	0.00	0.00											
EFM10	0.00	0.00	1.00	0.50	1.00	0.00	0.00	0.00	0.50	0.00	1.00											
EFM11	0.00	0.00	1.00	1.00	0.00	0.00	0.00	1.00	0.00	0.00	0.00											

The binary approach combines several essential advantages: a) modes are stored in binary format which dramatically reduces the memory usage, b) new modes are calculated from existing adjacent modes by using simple bitwise Boolean functions which are very fast compared to numeric operations, and c) the bitwise Boolean operations used are ‘exact’, hence, numerical accuracy problems are minimized.

### Gene regulatory information

Transcriptional regulatory networks (TRNs) control the process of gene expression in cells and, thereby how certain fluxes are activated or repressed. They determine how genes activate or repress certain fluxes. Hence, the gene regulatory information of a network imposes additional constraints on the reactions, and, as a consequence, has the potential to reduce the solution space resulting in a lower number of biologically feasible EFMs. Typically, the gene regulatory information is provided in form of Boolean functions [17], such as the NOT, OR, and AND operations. In what follows we will use a simple example to demonstrate the integration of TRNs into an EFM analysis.

As illustrated in Fig 1 we assume a TRN which only consists of a gene *GR* that activates reaction *R7r* and deactivates reaction *R9*. The function of gene *GR* can be transformed to a single Boolean expression: *R7r* = NOT(*R9*). This constraint means that the reaction *R7r* must not carry a flux when reaction *R9* carries a flux and vice versa.

A simple approach to get the reduced solution space is the application of this gene regulatory rule after all mathematically possible modes were calculated. Naturally, this method does not result in any performance improvement.

However, if we consider the basic principle of the binary approach described above, a dramatic speed up of the computation process can be obtained. The Boolean operation *R7r* = NOT(*R9*) implies that the rule is not obeyed if: a) *R9* = 1 = *t* and *R7r* = 1 = *t* or b) *R9* = 0 = *f* and *R7r* = 0 = *f*. The first expression is of particular interest, as it can be used to eliminate all modes from the solution matrix, *R*,—at any time of the iteration process—if *R9* and *R7r* do carry a flux. This statement is true, as a) the considered EFM itself disobeys the rule and b) all children EFMs generated from the parent EFM by combination with other EFMs will also disobey the rule. The latter property is owed to the fact that an active flux at a certain reaction will be retained by the bitwise OR operation for the rest of the computation procedure (see previous subsection for further details).

Removing a mode as soon as possible is of essential importance, as this mode is hindered to create offspring modes that would have to be eliminated at a later stage. The second expression (if *R9* = 0 = *f* and *R7r* = 0 = *f*) is of no use during the iteration process, as a *zero* flux value of *R9* or *R7r* can become a flux carrying reaction in a child mode that is created in a later iteration step. Hence, removing a currently disobeying mode with *R9* = 0 and *R7r* = 0 would result in the loss of children modes that obey the rule *R7r* = NOT(*R9*). However, the rule *R7r* = NOT(*R9*) for *R9* = 0 and *R7r* = 0 can still be used to remove infeasible modes after finishing the computation of all binary modes

The above considerations make clear that there are two types of situations: a) a rule can be applied during the iteration process and b) a rule can be applied during the post-processing step after finishing the mode calculation.

Determining if a Boolean rule *Ro* = ℬ(*R1*, …, *Rn*) qualifies for the iteration phase is simple. If the output reaction *Ro* of the rule is 0 (does not carry a flux) when all input reactions *R1*, …, *Rn* are 1 (carry a flux) then the rule can be used during the iteration phase.

Special care must be taken for reversible reactions, as they are split and, hence, occur twice in the extended set of reactions. If either the forward or the backward reaction carries a flux then the original reaction is supposed to be flux carrying when checked against a Boolean rule. Thus, in the example of Fig 1 the rule could also be written as *R9* = NOT(*R7f* OR *R7b*).

Applying these concepts to the example network with the gene regulatory rule *R7r* = NOT(*R9*) results in a mode matrix, *R*, after the first iteration step as shown in Table 5. Table 5 highlights in italic font style all reactions which disobey the rule *R7r* = NOT(*R9*). It can be seen that mode M5 disobeys the rule and is removed from the matrix, *R*.

**Table 5:** Mode matrix *R* after the first iteration step (left) converting reaction *R2* from numerical to binary form and after the last iteration step (right) for a regulated EFM analysis. After the final step *R* contains only binary values. Note that in the last step regEFMtool calculates fewer modes than the ordinary EFMtool (see Table 3). The italic font type highlights reactions disobeying the rule R7r = NOT(R9). After the first step M5 (highlighted in bold font) disobeys the iteration phase rule and is removed from the matrix. After the final step M8, M9, and M10 do not obey the iteration phase rule. Additionally, M1 and M7 (also highlighted in bold font) disobey the post-processing rule. M5 is also removed, as it is a futile 2-cycle mode created by splitting the reversible reaction R7r into two irreversible reaction.

	After first step										After last step										
	M1	M2	M3	M4	**M5**	M6	M7	M8	M9	M10	**M1**	M2	M3	M4	M5	M6	**M7**	**M8**	**M9**	**M10**	M11
R3	f	f	f	f	**f**	f	f	t	t	t	**f**	f	f	f	f	t	**t**	**t**	**f**	**f**	f
R4	f	t	t	t	**t**	t	f	t	t	f	**t**	t	t	t	f	t	**t**	**t**	**t**	**t**	t
R5	f	f	f	t	**t**	f	f	t	f	f	**f**	f	t	f	f	t	**f**	**f**	**t**	**f**	f
R6	f	f	t	f	**f**	f	f	f	f	f	**f**	t	f	f	f	f	**f**	**t**	**f**	**t**	f
R7f	f	f	f	f	***t***	t	t	f	f	f	***f***	f	f	t	t	f	***f***	***f***	***f***	***f***	f
R7b	t	f	f	f	**f**	f	t	f	f	t	***f***	f	f	f	t	f	***f***	***t***	***t***	***t***	t
R8	f	t	f	f	**f**	t	f	f	t	f	**t**	f	f	t	f	f	**t**	**f**	**f**	**f**	t
R9	f	f	t	t	***t***	f	f	t	f	f	***f***	t	t	f	f	t	***f***	***t***	***t***	***t***	f
R10	f	f	t	f	**f**	f	f	f	f	f	**f**	t	f	f	f	f	**f**	**t**	**f**	**t**	f
R11	f	f	f	t	**t**	f	f	t	f	f	**f**	f	t	f	f	t	**f**	**f**	**t**	**f**	f
R2	t	t	f	t	**f**	f	f	f	f	f	**t**	f	t	f	f	f	**f**	**f**	**t**	**t**	t
R1	-0.5	0.5	1.0	0.5	**0.5**	0.5	0.0	0.0	0.0	-0.5	**t**	t	t	t	f	f	**f**	**f**	**f**	**f**	f

In the next step mode M5 does not exist and, hence, fewer adjacency tests have to be performed. Table 5 shows the mode matrix R after the final iteration step. It can be seen that mode M8, M9, and M10 do not obey the iteration phase rule, as *R9* and *R7f* or *R7b* carry a flux. Hence, M8, M9, and M10 can be removed.

Furthermore, Table 5 illustrates that mode M1 and M7 disobey the post-processing rule, as *R9*, *R7f*, and *R7b* do not carry a flux value. Consequently, after removing the futile 2-cycle mode M5 the final mode matrix, R, only contains the five modes M2, M3, M4, M6, and M11. Before transforming the binary modes back to their numerical form the split irreversible reactions *R7f* and *R7b* must be combined to the reversible reaction *R7r*. The final set of feasible EFMs is listed in Table 4. Note that in comparison to the unregulated network six EFMs are biologically infeasible of which two are removed during the post-processing phase.

Often transcriptional regulation is not specified for both states of a reaction, e.g. a flux through *Ra* that represents the expression of a gene *Ga* might inhibit the flux of the reaction *Rb*, but no statement for reaction *Rb* might be possible if gene *Ga* is not expressed: *Rb* may or may not carry a flux [22]. Such a case cannot be expressed with the simple Boolean rule *Rb* = NOT(*Ra*), as this rule would require that *Rb* carries a flux if *Ra* does not.

In order to increase the flexibility of how rules can be formulated, we implemented a mathematical logic that knows three states: (i) *false*, (ii) *true*, and (iii) *undefined*. If the evaluation of a Boolean rule for a given set of input reactions yields the *undefined* state, then the rule is not consulted to constrain the solution space. The *undefined* state is introduced to the Boolean system by defining an *activity* for each reaction in all rules. A reaction can either be (i) 0-active, (ii) 1-active, or (iii) full-active. 0-activity means that the flux value is only considered if the reaction does not carry a flux. If a 0-active reaction carries a flux, then the *undefined* state is used for this reaction during the evaluation of the rule. If a reaction is 1-active then the flux value is only valid if the reaction does carry a flux. If a 1-active reaction does not carry a flux, then the *undefined* state is used. Full-active reactions exhibit defined values for both situations, when they carry a flux and when they do not. We denote the activity of rules by prefixing the characters ‘0’, ‘1’, and ‘f’ to the reactions, e.g. the full-active reaction *Rx* would be written as *fRx*.

Applying this concept to the above example would result in the following rule: *R7r* = NOT(*fR9*). If we assume 1-activity for *R9*, the rule is only considered if *R9* carries a flux and a statement for *R7r* can be made, as it is required that *R7r* is 0. However, if *1R9* does not carry a flux, the rule evaluates to *undefined* and the rule is not used. If we assume 1-activity rather than full-activity in the above example, we will find seven EFMs. This compares to five EFMs if we assume full-activity and eleven EFMs if no rules at all are used (see Table 4).

Note that one single reaction might appear within a rule multiple times with any of the three defined activities. If more than one rule is specified, each of the rules must be obeyed by a tested mode. Hence, the rules of a given rule set can be considered as combined by Boolean AND operations. If—due to network compression—reactions are combined with other reactions, we apply the same transformation to the rule set as well. For instance, rather than R7r = NOT(fR9) we use a rule acting on the compressed reaction R7r_compressed = NOT(fR9). This is possible as the compression provides a bijective transformation, that is R7r = 0 if and only if R7r_compressed = 0. (The same one-to-one correspondence applies for “1” and “undefined”.) For further details regarding our implementation of the three-state logic and Boolean rules see [23].

### Implementation

We implemented our approach as an extension to the open source software *efmtool*[6]. The mode elimination algorithm was realized by adding three *Java* packages to the original version of *efmtool*. The three packages contain ten new *Java* classes. These new classes are responsible for handling the genetic rules and checking the modes against them. Two already existing *Java* classes were slightly enhanced in order to invoke the mode check. The Boolean rules are provided by an additional input file using the command line argument -generule. The extended version of *efmtool* was compiled by *JDK 1.7.0*. The implementation of the extension was performed as non-invasive as possible, which means that the performance gain might be even better if the new method is integrated to *efmtool* more thoroughly. The mode checks for the iteration phase were implemented using binary bit patterns where the patterns are created simply by setting the involved reactions (all input reactions and the output reaction) to 1. Note that *efmtool* uses an inverse logic where 0 stands for flux carrying reactions and 1 stands for not carrying a flux. Hence, the involved bitwise operations and comparison have to be changed accordingly. If a tested mode has every bit set that occurs in the binary bit pattern of a rule then the mode is eliminated. The mode check for the post-processing step was realized by utilizing a reverse polish notation approach that allows a simple and fast execution of Boolean functions with any values for the input reactions. The general sequence of operation of our extended version of the binary DDM is shown in S1 Table.

## Results

A brief introduction to and an initial test of our approach can be found in [23], where we used the *E. coli* core model by [17] and gene regulatory information in form of a gene-enzyme-reaction mapping [24]. The used rule set consisted of 58 Boolean functions of which just four rules were iteration phase rules. The results in [23] showed that the performance gain in terms of memory usage and execution time is mainly caused by the four iteration phase rules. In the present work, we study the effects of iteration phase gene rules on the number of adjacency candidates, intermediate modes, and on the obtained performance gain in greater detail.

We used the medium sized metabolic yeast model by [25] and transcriptional rules by [26]. The model consisted of 218 metabolites and 230 reactions (of which 197 were irreversible) in two compartments (cytosolic and mitochondrial). An SBML description of the model can be found in S1 File. The gene-enzyme-reaction mapping used to exclude infeasible EFMs contained four Boolean functions which are listed in Table 6. The rules consider four genes (ACH1, ICL1, MDH1 and MDH2) which are glucose repressed in *S. cerevisiae*[26]. This means, in terms of the model, that when *R_GLCt1* is active, the reactions corresponding to the above genes must be inactive. Five reactions participate in these four rules. We did not consider any post-processing rules in our analysis, as the post-processing rules are merely a simple filtering procedure applied after the iteration phase. The performance gain obtained by post-processing rules mainly depends on the number of modes that are eliminated by those rules. Post-processing rules have (i) no effect on the required main memory, (ii) a minor effect on the execution, and (iii) a strong effect on the hard disk space if many modes are eliminated [23].

**Table 6:** The four Boolean rules, *X* = NOT(1*R*_*GLCt*1), used by the introduced elimination algorithm to exclude biologically infeasible EFMs during the iteration phase. R_GLCt1 denotes the glucose uptake transporter. R_GLCt1 is considered to be 1-active, i.e. X = 0 if R_GLCt1 = 1 and undefined otherwise. Gene regulatory information for this model was taken from [26].

Rule	*X*	Gene	Function
GR1	R_ACOAH	ACH1	Acetyl-CoA hydrolase
GR2	R_ICL	ICL1	Isocitrate lyase
GR3	R_MDH	MDH2	Malate dehydrogenase
GR4	R_MDHm	MDH1	Malate dehydrogenase (mitochondrial)

Table 7 compares a regular run without regulatory information and a run using the available gene regulation rules. Both runs were performed on a Linux Ubuntu 12.04 computer with 2 Intel Xeon CPUs (6 cores each) and a total of 192 GB of RAM using 10 parallel threads. The table shows that after the iteration phase 92.4 million modes were obtained without regulatory information. Using all four Boolean rules resulted in a mode reduction by a factor of more than 200 and resulted in 453,563 modes. This mode removal during the iteration phase caused a reduction of the iteration runtime from 54.5 hours to 24.7 minutes and a decrease of the maximum number of adjacent candidates from 1.5 ⋅ 10^15^ to 1.4 ⋅ 10^11^. This huge reduction of the total number of modes had a major influence on the required hard disk space which was decreased from 357.4 GB to 1.7 GB when an uncompressed double precision text format was used. Table 7 clearly shows that considering gene regulatory information in the computation process has a huge impact on the computational key properties of the calculation of EFMs.

**Table 7:** Comparison of EFM calculation with and without taking into account gene regulatory information. The required disk space is given for a result file containing all modes in text format using double precision. The line ‘max. adjacent candidates’ shows the maximum number of potentially occurring adjacent pairs.

	w/o gene regulation	with gene regulation
No. of modes	92.4 ⋅ 10^6^	0.45 ⋅ 10^6^
Max. adjacent candidates	1.5 ⋅ 10^15^	1.4 ⋅ 10^11^
Max. RAM usage	161 GB	1.1 GB
Runtime	54.5 h	0.41 h
Disk space	357.4 GB	1.7 GB

In order to verify that the presented extension of the *efmtool* computes the correct EFMs, an extra software tool was developed that applies the Boolean rules to the complete and unfiltered set of EFMs in a post-processing step. The two tools computed identical sets of EFMs ensuring that the *efmtool* extension produces the correct result.

Table 8 shows the development of the number of intermediate modes as a function of finished iterations. The number of intermediate modes mainly determines the required amount of RAM. In total, 47 iteration steps were performed in order to compute the complete set of EFMs. Up to iteration 4 not a single EFM was eliminated and the inclusion of gene regulatory information had no effect. The first removal occurred at iteration 5, where 11 modes were deleted. Although in total only 0.91 million modes were removed during the iteration phase, the final number of modes was reduced by 91,98 million modes. This huge reduction is a result of lost parent modes which otherwise could have spawned a multitude of new children. The large difference of intermediate modes (1.7 million with regulation vs. 209 million modes without regulation) has a direct impact on the required RAM which is just 1.1 GB for the run with gene regulation compared to 161 GB for the case without regulation.

**Table 8:** Comparison of the number of intermediate EFMs as a function of the iteration step for simulations with gene regulatory information (rule GR1, GR2, GR3, and GR4) and without gene regulatory information.

Iteration No.	No. of removed infeasible modes	No. of modes incl. gene regulation	No. of modes w/o gene regulation
1	0	66	66
2	0	70	70
3	0	75	75
4	0	85	85
5	11	122	133
6	1	119	142
7	0	377	468
8	0	1625	2,175
9	0	2,529	3,359
10	1,397	13,467	20,316
11	13,764	35,327	78,937
12	3,921	62,373	158,870
13	1,166	122,144	331,088
14	3,292	90,172	352,506
15	1,963	129,289	530,164
16	5,495	176,374	803,112
17	9,533	281,685	1,498,951
18	47,530	256,339	1,830,652
19	488	317,390	2,305,473
20	1,171	219,660	2,308,759
21	0	211,660	2,253,813
22	66,394	96,340	1,491,202
23	0	81,745	1,470,314
24	0	89,665	1,481,940
25	0	113,156	2,143,606
26	0	95,962	2,203,512
27	0	129,159	2,668,814
28	0	209,405	4,454,288
29	0	209,405	5,370,688
30	0	209,405	6,034,256
31	0	289,852	7,413,300
32	0	289,852	7,413,300
33	0	289,852	8,050,776
34	0	356,188	10,693,536
35	0	582,988	17,777,572
36	0	682,332	28,376,662
37	0	754,827	30,042,830
38	754,777	410,403	49,312,818
39	0	635,137	75,527,292
40	0	635,137	82,370,868
41	0	826,802	108,752,476
42	0	1,655,574	209,418,474
43	1	437,237	58,830,104
44	0	437,237	58,830,104
45	0	355,714	63,325,875
46	0	355,714	72,412,386
47	0	453,590	92,433,694
sum	910,903		

**Fig 2:**
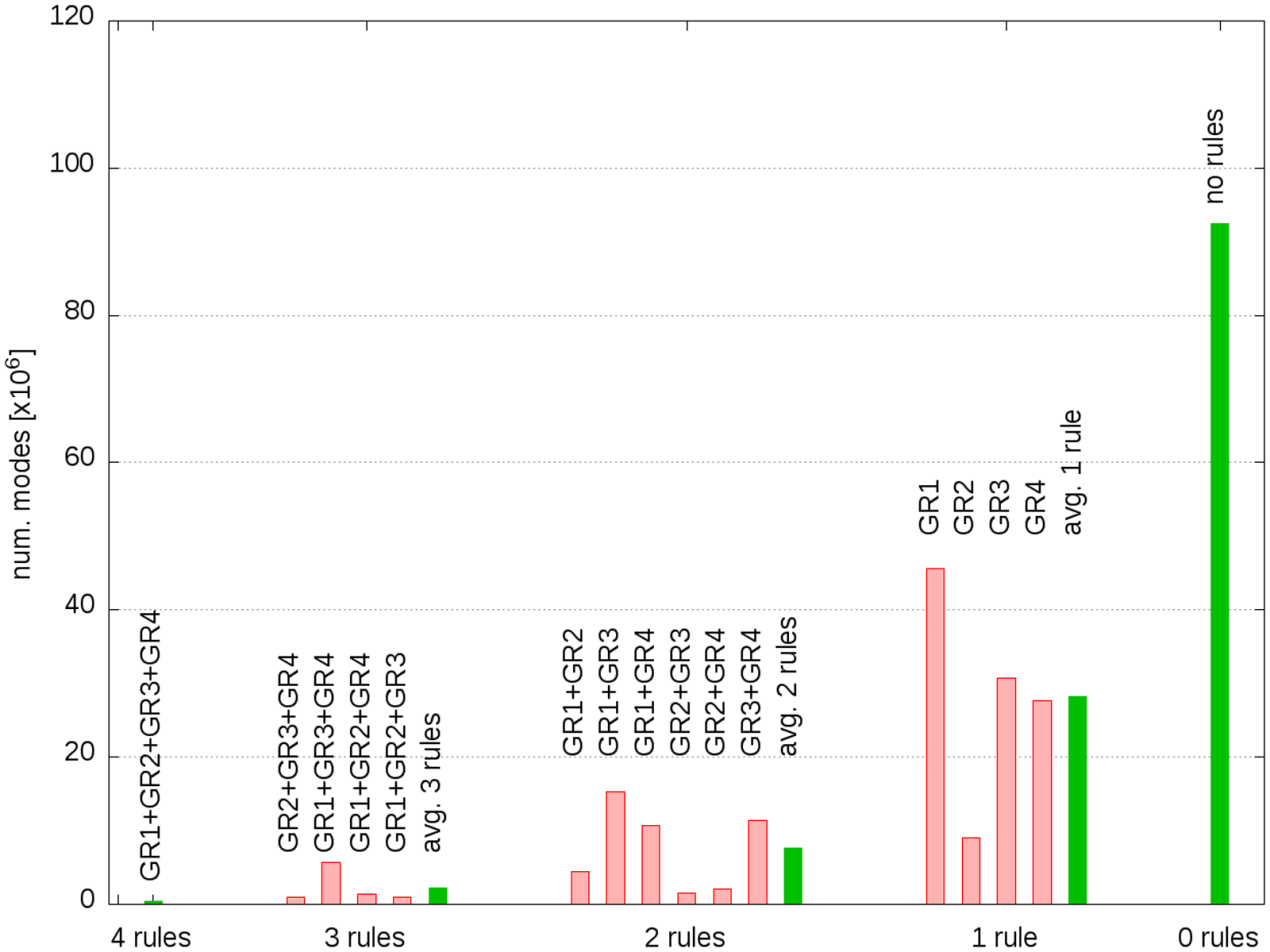
Number of modes as a function of all possible combinations of the four iteration phase rules. The green bars show the average mode number for cases with one, two, or three gene rules.

In order to study the effect of varying number of iteration phase rules on the execution time, we performed runs with all possible combinations of the four used iteration phase rules. Fig 2 depicts the number of modes as a function of gene rules. The figure clearly shows that the number of modes increases with decreasing number of rules. The red bars show the results of the individual runs for cases with one, two or three iteration phase rules, whereas the green bars show the averaged results for these cases.

Similar to the decrease in the number of EFMs we found that the average execution time extremely decreases with increasing number of rules, too (see S1 Fig). Fig 2 and S1 Table show data of runs that used 10 parallel threads. Although the gain of execution times of the individual runs vary significantly, the average values clearly illustrate the performance gain obtained by using iteration phase rules.

In order to find an initial value of the mode matrix, *R*, the kernel of the extended stoichiometric matrix is computed. Before the iteration phase is started the reactions/rows of the kernel matrix *K* are sorted. This is done by putting all reactions with only positive values to the top (e.g. see Table 2), which results in the maximum number of reactions that can be transformed to the binary form before the iteration procedure is even started.

The performance of the DDM is very sensitive to the order of the reactions in the Kernel matrix, *K*[19]. Several approaches can be applied to sort those reactions that also contain negative values, e.g. taking no special measures (random order), ordering by increasing potential adjacency candidates (number of negative values times number of positive values), and various dynamic re-ordering methods [19, 21, 27]. As the iteration phase rules can only be applied if the involved reactions are already converted to the binary representation, it seems beneficial to convert all reactions—that are involved in iteration phase rules—to the binary form as soon as possible. We illustrate this point by means of an example. Structure and details of the example network used may be found in S2 Fig and S6, S7 and S8 Tables.

Table 9 shows the standard re-ordering of reactions in increasing order of adjacency candidates for the example network in S2 Fig Suppose that the reactions R1 and R5 are involved in a single iteration phase rule, e.g. *R*1 = NOT(*fR*5). Thus R1 and R5 should get a higher priority. Table 9 shows the effect of rearranging the reaction order. Note that the position of a reaction is not changed if it is already converted to its binary form before the start of the iteration phase.

**Table 9:** Effect of re-arranging the order of reactions for the simple example shown in S7 Table because of a gene rule that involves reaction *R*1 and *R*5.

Initial order before execution	Sorted by adjacency candidates	Number of adjacency candidates	Re-ordered	Number of adjacency candidates
R1	R3	0	R3	0
R2	R6f	0	R6f	0
R3	R6b	0	R6b	0
R4f	R7f	0	R7f	0
R4b	R7b	0	R7b	0
R5	R8	0	R8	0
R6f	R9	0	R9	0
R6b	R10	0	R10	0
R7f	R4b	0	R4b	0
R7b	R11b	0	R11b	0
R8	R12f	0	R12f	0
R9	R12b	0	R12b	0
R10	R11f	2	**R5**	4
R11f	**R5**	4	**R1**	6
R11b	**R1**	6	R11f	2
R12f	R2	6	R2	6
R12b	R4f	12	R4f	12

In order to investigate the effect of re-ordering the reactions on the program runtime, we used the *S. cerevisiae* model and the rules listed in Table 6 and performed two sets of runs: (i) runs with a standard *efmtool* reaction order (by increasing number of potential candidates) (ii) runs with a rearranged order of the reactions to allow for an early conversion of the involved reactions to binary form. The re-ordering was obtained by moving all reactions involved in rules directly behind the set of reactions that have only positive values. The number of reactions of the used yeast model after the initial compression step of the *efmtool* was 112, of which 65 reactions only contained positive values and, hence, could be binarized prior to the iteration procedure. Consequently, during the iteration phase 47 steps had to be executed. Therefore, only reactions at a position larger than 65 were rearranged. Note that a single rule with just two participating reactions, such as *R*1 = NOT(*fR*5) may involve more than two reactions during the iteration process, as (i) an involved reaction might be reversible and is split in the pre-processing phase and (ii) the network compression algorithm maps the original reaction to multiple compressed reactions. Table 10 lists the key properties of the reactions regarding the re-ordering approach. It can be seen that the rules GR1 and GR4 only involved reactions at position indices which were less or equal to 65. Hence, no re-ordering was required for these rules. The rules G2 and G3 involved reactions larger than 65 and, hence, they were subject to re-sorting. The reactions at position 69, 82, 86, 102 were moved to the positions 66, 67, 68, and 69, respectively, corresponding to the iterations 1, 2, 3, and 4.

**Table 10:** Key properties of reactions participating in iteration phase rules regarding the re-ordering approach. A reaction is subject to re-ordering if the position index after splitting, compression, and initial sorting is larger than 65.

Reaction	Rule	Num. reactions after splitting/compression	Position after splitting/compression
R_GLCt1	GR1, GR2, GR3, GR4	3	12, 20, 65
R_ACOAH	GR1	2	30, 31
R_ICL	GR2	3	69, 82, 86
R_MDH	GR3	2	46, 102
R_MDHm	GR4	1	11

**Fig 3:**
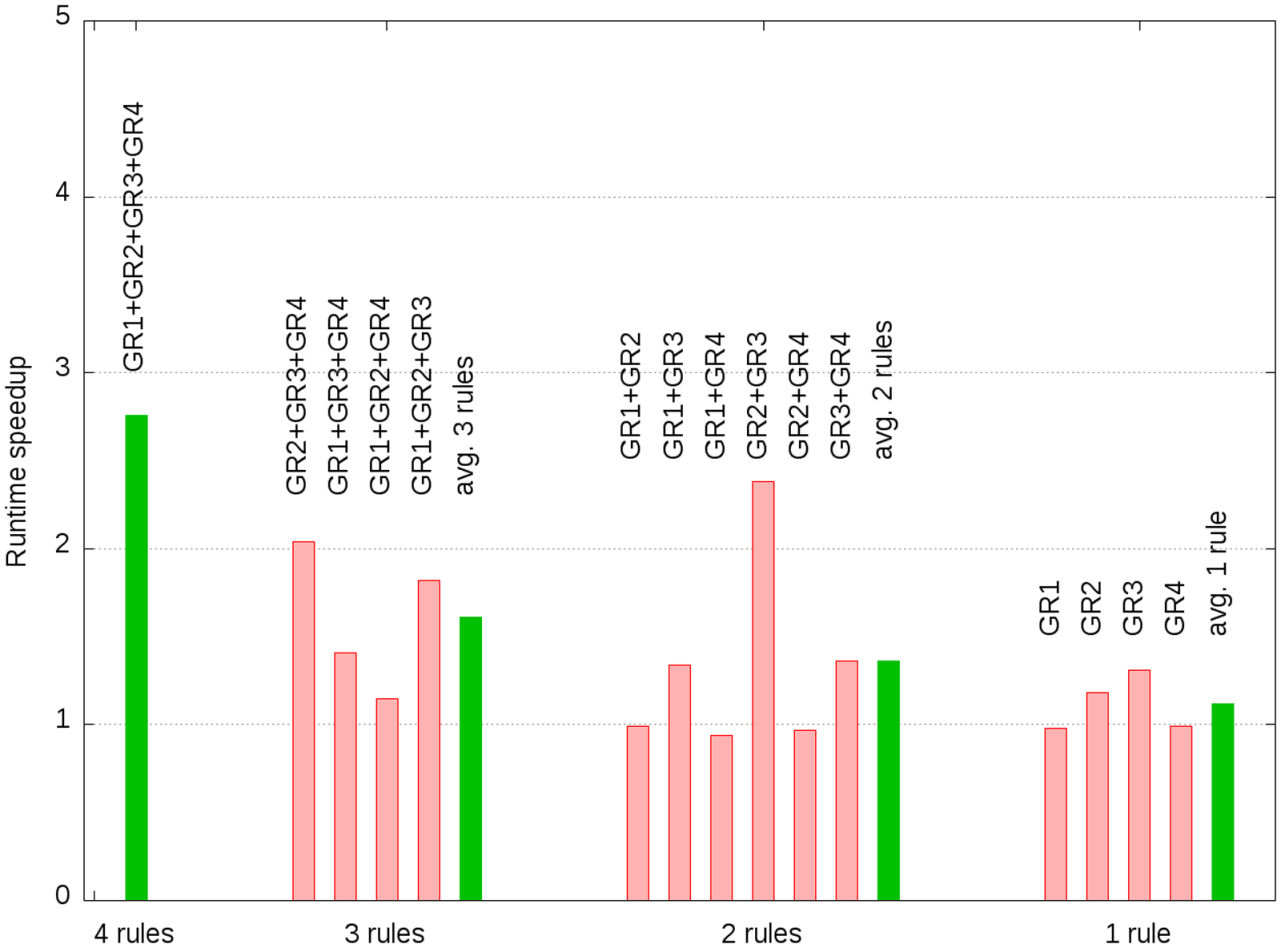
Runtime speedup (ratio of execution time without resorting to execution time with resorting reactions) as a function of used gene rules.

Fig 3 shows the speedup of the execution time as a function of used gene rules. The speedup was defined as the ratio of the execution time without resorting to the execution time with resorting the reactions. As shown in Fig 3 the maximum speed up was achieved when all four gene rules were used in which case the program run 2.76 times faster when the reactions were resorted before the iteration phase. Furthermore, it can be seen that the runtime improvement decreased with a decreasing number of used iteration phase rules. If only GR1 or GR4 were used no runtime gain could be achieved, as no re-ordering was performed for those two rules.

**Fig 4:**
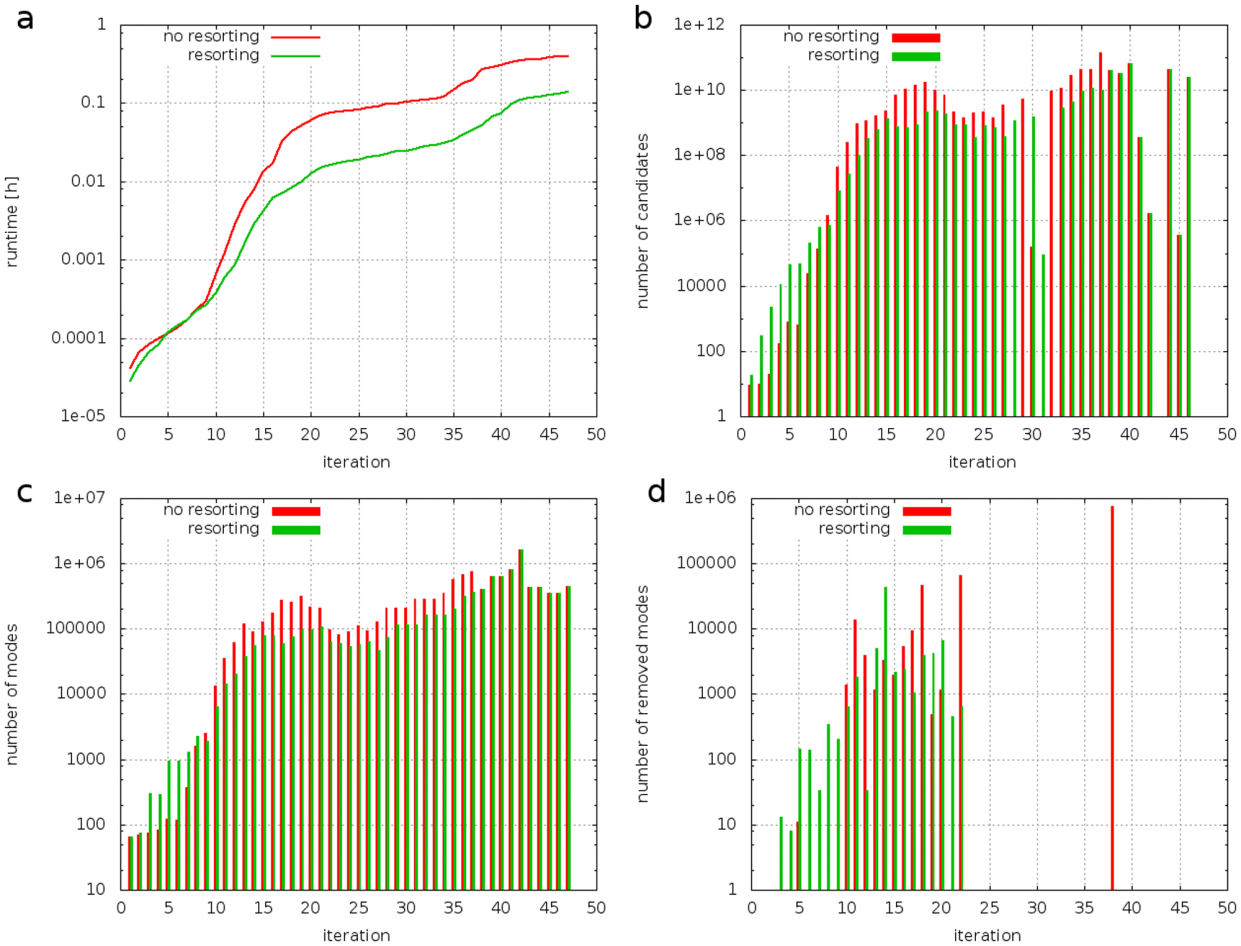
Comparison of runs with (green) and without (red) resorting the reaction order for a case with four rules (GR1, GR2, GR3, and GR4). The diagrams show the accumulated runtime (a), the number of adjacency candidates (b), the number of intermediate modes (c), and the number of modes eliminated by the rules (d) as a function of the iteration step.

As can be seen in Fig 4a the execution time dropped by a factor of 2.76 from 0.41 to 0.15 hours if all four rules were used and re-reordering was applied. Our analysis showed that re-sorting the reactions results in an increased number of eliminated modes during the first iteration steps. The elimination of the modes reduced the number of intermediate modes during the iterations 10 to 37 (Fig 4c), caused by the reduced number of adjacent candidates (Fig 4b). Fig 4d shows the effect of rule GR3 which removed more than 0.75 million modes at iteration 47, corresponding to the reaction index 102 (see Table 10). When reaction re-ordering was applied all those 0.75 million biologically infeasible modes were already eliminated at previous iterations and, hence, reduced the total number of intermediate modes that had to be processed and decreased the total number of adjacency tests that had to be performed. Note, however, that a significant reduction of modes only occurs if the rule is disobeyed by enough intermediate modes. If a rule only removes a small number of modes, the mode elimination might not outweigh the negative effects of creating more adjacency candidates and intermediate modes at early stages of the procedure and, consequently, might result in longer runtimes if reaction re-ordering is applied. This is of considerable relevance, as it is not known *a priori* how effective an iteration phase rule is going to be. Even though our analysis only considers an isolated case, the results show that—in general—finding the optimal reaction order is not trivial and indicate that re-sorting the reactions might have a positive effect on the runtime.

In order to further show the capability of our approach, we performed three simulations for an *E. coli* core model augmented by amino acid metabolism. The extended model consisted of 178 metabolites and 209 reactions (84 reversible). An SBML description of the model can be found in S2 File. The simulations were performed on a Linux Ubuntu 12.04 computer with 2 Intel Xeon CPUs (12 cores each) and a total of 384 GB of RAM using 20 parallel threads. Without using gene regulatory information the simulation was terminated by the operating system after 29 days, as the program requested more than the available 384 GB of RAM. The simulation reached 37 of a total of 39 iterations. At that point more than 702 million intermediate modes had been calculated indicating that the final set of EFMs consists of considerably more than 1 billion elements. If the four gene rules reported in [23] were included the entire simulation could be finished in just over 61 hours and resulted in 185 million EFMs. Activating the reaction resorting by the command line option -rulesort true reduced the execution time by additional 4 hours.

Applying the four regulatory rules listed in Table 6 restricted the metabolic capabilities of the organism. In the following analysis we will look at the global effects of the gene rules on other reactions in the network. Note that these effects were consequences of the network structure and not of the rules *per se*. A summary of the most significant changes was listed in Table 11.

**Table 11:** Changes in the reaction activity frequency with ($\alpha_{i}^{\text{w}}$) and without ($\alpha_{i}^{\text{w/o}}$) applying the four GRs listed in Table 6. *α*_*i*_ is defined as the frequency of an active reaction *i* in all EFMs. For simplicity we only listed reactions with $|\alpha_{i}^{\text{w}}-\alpha_{i}^{\text{w/o}}|>10\%$.

Rule	Reaction	Gene	$\alpha_{i}^{\text{w/o}}$ (%)	$\alpha_{i}^{\text{w}}$ (%)	$\alpha_{i}^{\text{w/o}}$—$\alpha_{i}^{\text{w}}$ (%point)
GR1	R_ACOAH	ACH1	50.70	0.00	-50.70
GR2	R_ICL	ICL1	90.17	0.00	-90.17
GR3	R_MDH	MDH2	66.81	0.00	-66.81
GR4	R_MDHm	MDH1	70.09	0.00	-70.09
	R_ACS	ACS2	49.30	100.00	50.70
	R_ICDHy	IDP2	42.27	62.26	19.99
	R_PYRt2m	–	77.52	94.10	16.58
	R_ACt2r	BPH1	31.18	45.88	14.70
	R_H2Otm	–	60.60	75.20	14.60
	R_GLYCt	FPS1	86.51	100.00	13.49
	R_G3PT	GPP1, GPP2	86.52	100.00	13.48
	R_TKT2	TKL1	40.97	54.22	13.25
	R_GLUSxm	GLT1	83.25	95.51	12.26
	R_GLNt2m	–	83.25	95.51	12.26
	R_ICDHxm	IDH1, IDH2	70.32	81.17	10.85
	R_CSm	CIT1, CIT3	87.88	77.41	-10.47
	R_PIt2m	MIR1	95.45	80.61	-14.84
	R_SUCCt2r	–	28.36	0.00	-28.36
	R_SUCFUMtm	SFC1	38.44	0.00	-38.44
	R_SUCCtm	DIC1	42.25	0.00	-42.25

GR2 states that isocitrate lyase R_ICL (gene ICL1) is inactive under high glucose conditions, shutting down the glyoxylate cycle. As a consequence two succinate transporters were disabled: R_SUCFUMtm (gene SFC1, a succinate—fumarate antiporter between the cytosol and the mitochondrion) and R_SUCCtm (gene DIC1, which catalyzes a dicarboxylate-phosphate exchange across the mitochondrion). Both transporter are known to be tightly co-regulated with the glyoxylate cycle and their predicted inactivity under high glucose conditions is in accordance with experimental data [28]. Note that with inactive DIC1 and SFC1 an uptake of succinate is impossible, too. Under this condition our analysis revealed that no EFM was able to metabolize extracellular succinate.

The used metabolic model contains four biochemical reactions for the production of acetyl-CoA [mitochondrial pyruvate dehydrogenase (R_PDHm, genes PDE1 to PDE3), cytosolic and mitochondrial acyl-CoA synthetase (R_ACS, gene ACS2 and R_ACSm, gene ACS1, respectively), and acetyl-CoA hydrolase (R_ACOAH, gene ACH1)]. In the unregulated case none of theses reactions is essential. However, when acetyl-CoA hydrolase (R_ACOAH, gene ACH1) was inactivated (GR1) all biomass producing EFMs needed an active cytosolic acyl-CoA synthetase (R_ACS, gene ACS2). This prediction is in agreement with experimental findings that ACS2 is essential for growth on glucose [29].

In addition we found that if the cytosolic or the mitochondrial malate dehydrogenase was deactivated (GR3 or GR4), glycerol-3-phosphate phosphatase (R_G3PT, genes GPP1 and GPP2) became essential for growth—in contrast to experimental evidence [30]. That finding highlighted a deficiency of the metabolic model, as an inactive malate dehydrogenase has the effect that in the model NAD regeneration is only possible via the production of glycerol.

## Discussion

Currently, extensive transcriptional regulatory information is available only for a limited number of organisms. In particular this is true for continuous regulatory models such as differential equation models [15]. However, growing scientific effort is put into the investigation of transcriptional regulatory mechanisms. In recent years regulatory models for more and more organisms have been developed, e.g. *Escherichia Coli*[24], *Arabidopsis thaliana*[31], *Drosophilia melanogaster*[32], and *Saccaromyces cerevisiae*[26, 33, 34]. Consequently, it can be expected that in the near future our approach will not suffer from the unavailability of regulatory information any longer. Most important, our method does not require knowledge of the complete transcriptional network of the organism as even an incomplete data set of regulatory information can have a huge positive influence on the execution time. Our study shows that just four simple rules not only have a huge effect on the runtime and RAM consumption, but also improve the predictive quality of the model. This is not only true for the set of rules but for each individual rule as well as. However, note that not all rules can be used for speeding up the enumeration process. “Iteration phase rules” have to evaluate to zero, while all its input arguments are logical one. This particular structure guarantees that our algorithm does not erroneously identify feasible EFMs to be infeasible. The simplest form of such a rule is a NOT statement, e.g. the condition specific repression of the glyoxylate shunt *E. coli*, *Rhodobacter capsulatus*[35], *Streptomyces collinus*[36], or *Paracoccus versutus*[37] is of such a form and can be used as an iteration phase rule. The examples above show that simple iteration phase rules can be found in various, even less well studied organisms too.

As described above, the used binary nullspace algorithm heavily relies on the fast execution of bit vectors. Naturally, the presented method does not allow to access continuous values of the computed fluxes during the iteration phase. Consequently, our approach cannot gain from the use of continuous regulatory models, such as linear models and differential equation models [14], which describe the system state of the regulatory network with continuous values. However, regulatory effects are often sigmoidal and, hence, can be sufficiently modeled by discrete systems [38]. Boolean regulatory networks have been successfully combined with other metabolic modeling applications, such as flux balance analysis [22, 39]. As EFMs are minimal pathways under steady state, dynamic regulatory rules which consider time dependent effects on the relationships between genes cannot be incorporated easily into our approach.

In general, the number of modes of metabolic networks is very high. By using a novel approach of parallel execution the authors of [8] were able to compute all 1.9 billion EFMs of a *Phaeodactylum tricornutum* model that consisted of 318 reactions and 355 metabolites. It requires approximately 1.1 TB to save these 1.9 billion EFMs in binary format to a hard disk. Consequently, storing, handling, analyzing, and using such a huge number of EFMs is computationally extremely challenging. Several approaches try to reduce the numerical effort by, for instance, computing only the binary projection of EFMs in subsystems of interest [40], or by representing the complete set of EFMs by a (randomly drawn) subset of EFMs [41–44]. In fact, it has been suggested that only very few EFMs are physiologically relevant [45]. Thus, it would be desirable to identify only those EFMs. Here we presented a method which achieved exactly that. By using transcriptional regulatory rules we efficiently eliminated only biologically infeasible EFMs. Thus, rather than randomly reducing the number of EFMs our approach implements a reduction method based on biological knowledge. By applying available biological knowledge we were also able to detect new information, as demonstrated in the case of acyl-CoA synthetase and glycerol-3-phosphate phosphatase. The former was correctly identified to be essential while the latter identified gaps in the used model. Note however, that all findings are independent of any optimization principle commonly used in approaches that are based on flux balance analysis (FBA), as EFMs characterize the full solution space. Hence, we may also use our approach to detect so far unknown regulatory rules. Suppose we do not know about the down regulation of acetyl-CoA hydrolase during growth on glucose but rather only know that a knockout of ACS2 causes a growth phenotype. That is, we are interested in candidate genes which—if repressed—eliminate growth. The question is essentially a metabolic engineering problem where the aim is to eliminate undesired metabolic capabilities. Optimal targets can be identified based on an EFM analysis by calculating minimal intervention sets removing all undesired states [46, 47]. These targets represent potential regulatory switches allowing for an iterative discovery of new regulatory rules and triggering further experiments. In principle we may also use FBA to derive these predictions. While gene essentiality can easily be checked by FBA, predicting for instance synthetic lethal pairs is computationally challenging. Due to the explosion of possible combinations detecting synthetic lethality in groups of four or more is impossible to do in an FBA framework. Here an EFM analysis in combination with efficient methods for calculating minimal cut sets is currently the only feasible option [48, 49]. In fact, based on our (regulated) EFM analysis we computed 22,533 synthetic lethalities in groups of up to six reactions with a modified hitting set algorithm [50] in less then two hours (see S3 File).

We used Boolean rules to describe transcriptional regulation. Although Boolean regulation has successfully been coupled with flux balance analysis [33, 51] it is plagued by two major problems: (i) Boolean rules sometimes over-restrict metabolic models [52], and (ii) a Boolean description of transcriptional networks is dependent on the specific problem representation [22]. That is, the feasible metabolic network states are different if the transcriptional network is described by explicit rules (where each state of a gene can be calculated explicitly from the states of all other input data) or implicit rules. However, our activity based three-value logic introduced in this work is a more general way of expressing transcriptional networks in a mathematical formulation. Our approach is able to represent explicit rules, implicit rules, and a rule set which less restrictive than either of the two alternative formulations (see S4 Table). Thus compared to standard approaches our activity based formulation can be used to implement extremely conservative rules and, therefore, is less prone to over-restriction.

We implemented a novel approach to speed up the computation of EFMs of a metabolic network by extending the open source program *efmtool* written by Marco Terzer. Our extension allows the consideration of gene-enzyme-reaction mappings in the process of the EFM calculation. Consequently, our method computes the complete set of EFMs with the exception of all modes that are detected to be biologically infeasible because they disobey the provided gene regulatory rules. The biologically infeasible flux modes are constantly eliminated during the calculation process. By implementing an early stage exclusion of modes a dramatic reduction of computational costs was achieved which pushes the maximum size of computable networks to new and higher limits. In a brief introduction we successfully tested our approach with *E. coli*[23]. In this work we presented a detailed description of our method and an elaborate analysis of the effect of gene regulatory rules using a medium-scale yeast model. We think that our approach is another step to the final goal of studying genome-scale metabolic networks by elementary flux modes.

## Supporting Information

S1 Fig Execution time of *regEfmtool* as a function of all possible combinations of the four iteration phase rules. The green bars show the average execution time for cases with one, two, or three gene rules.
(TIFF)

S2 Fig Example network used to demonstrate reaction-reordering due to gene regulatory information. The network consists of five metabolites and twelve reactions of which five are reversible and, hence, split in non-reversible forward and backward reactions. The stoichiometric matrix of the example is shown in S6 Table. The network has 30 elementary flux modes if no gene rules are used to restrict the solution space (see S8 Table). If the rule *R*1 = (!*fR*5) is applied, the network contains only eleven modes.
(TIFF)

S1 Table Stoichiometric matrix, *S*, of the example network. External metabolites which are irrelevant for the calculation of the elementary flux modes are omitted.
(PDF)

S2 Table Extended stoichiometric matrix, *S*_ext_, of the example network shown in Fig 1 after splitting the reversible reaction R7r into the two irreversible reactions R7f and R7b. (PDF)

S3 Table Binary representation of all elementary flux modes of the example network shown in Fig 1. 1 means that the reaction carries a flux and 0 means the reaction carries no flux. Note that the futile two-cycle of the reversible reaction *R7r* has already been removed and the forward and backward irreversible reactions (*R7f* and *Rfb*) have been combined to the reversible reaction *R7r* by a bitwise OR operation.
(PDF)

S4 Table Comparison of four methods of genetic rule formulations: (i) explicit by Jensen et al., (ii) implicit by Jensen et al., (iii) three state logic—implicit functionality, and (iv) three state logic—direct implementation. The values of the reactions are shown in columns mig1, mth1, gln, rgt1. The characters ‘⇒’ and ‘⇔’ denote the explicit and implicit formulation as defined by Jensen et al. Feasible combinations of reactions are highlighted in color. For the *undefined* state of the three-state logic the character ‘u’ is used.
(PDF)

S5 Table Pseudocode for the *regEFMtool*. Text sections in *italic* style indicate our modifications compared to the binary method reported by Gagneur and Klamt [5].
(PDF)

S6 Table Extended stoichiometric matrix S_*ext*_ of the example network shown in S2 Fig. (PDF)

S7 Table Mode matrix *R* of a simple example network after sorting the reactions and before executing the iteration phase. (PDF)

S8 Table The complete set of elementary modes of the example network shown in S2 Fig if no gene rules are used to restrict the solution space. (PDF)

S1 File SBML file of the used yeast model consisting of 218 metabolites and 230 reactions (197 irreversible) in two compartments. (SBML)

S2 File SBML file of the used *E. coli* core model consisting of 178 metabolites and 209 reactions (84 irreversible). (SBML)

S3 File List of synthetic lethality in groups of up to six reactions for the extended *E. coli* network used in this work. (BZ2)

S4 File Used version of the software *regEfmtool*. (GZ)

## References

[1] Schuster S, Fell DA, Dandekar T (2000) A general definition of metabolic pathways useful for systematic organization and analysis of complex metabolic networks. Nat Biotech 18: 326–332. 10.1038/73786 10700151

[2] Schuster S, Dandekar T, Fell DA (1999) Detection of elementary flux modes in biochemical networks: a promising tool for pathway analysis and metabolic engineering. Trends in Biotechnology 17: 53–60. 10.1016/S0167-7799(98)01290-6 10087604

[3] Zanghellini J, Ruckerbauer DE, Hanscho M, Jungreuthmayer C (2013) Elementary flux modes in a nutshell: Properties, calculation and applications. Biotechnology Journal 8: 1009–1016. 10.1002/biot.201200269 23788432

[4] Klamt S, Stelling J (2002) Combinatorial complexity of pathway analysis in metabolic networks. Molecular Biology Reports 29: 233–236. 10.1023/A:1020394300385 12241063

[5] Gagneur J, Klamt S (2004) Computation of elementary modes: a unifying framework and the new binary approach. BMC Bioinformatics 5:175 10.1186/1471-2105-5-175 15527509 PMC544875

[6] Terzer M, Stelling J (2008) Large-scale computation of elementary flux modes with bit pattern trees. Bioinformatics 24: 2229–2235. 10.1093/bioinformatics/btn401 18676417

[7] Jevremovića D, Trinh CT, Srienc F, Sosad CP, Daniel B (2011) Parallelization of nullspace algorithm for the computation of metabolic pathways. Parallel Computing 37: 261–278. 22058581 10.1016/j.parco.2011.04.002 PMC3205353

[8] Hunt KA, Folsom JP, Taffs RL, Carlson RP (2014) Complete enumeration of elementary flux modes through scalable demand-based subnetwork definition. Bioinformatics 30: 1569–78. 10.1093/bioinformatics/btu021 24497502 PMC4029027

[9] von Kamp A, Schuster S (2006) Metatool 5.0: fast and flexible elementary mode analysis. Bioinformatics Application Note 22: 1930–1931. 10.1093/bioinformatics/btl267 16731697

[10] Klamt S, Saez-Rodriguez J, Gilles ED (2007) Structural and functional analysis of cellular networks with cellnetanalyzer. BMC Systems Biology 1:2 10.1186/1752-0509-1-2 17408509 PMC1847467

[11] Trinh CT, Thompson RA (2012) Elementary mode analysis: A useful metabolic pathway analysis tool for reprograming microbial metabolic pathways. In: Wang X, Chen J, Quinn P, editors, Reprogramming Microbial Metabolic Pathways, Dordrecht: Springer Netherlands, volume 64 pp. 21–42. 10.1007/978-94-007-5055-5_2 23080244

[12] Open Source Initiative (2012) The BSD License. http://www.opensource.org/licenses/BSD-2-Clause.

[13] ETH Zurich, Computational Systems Biology Group (2012) efmtool—Elementary Flux Mode Tool. http://www.csb.ethz.ch/tools/efmtool.

[14] Schlitt T (2013) Approaches to modeling gene regulatory networks: A gentle introduction. In Silico Systems Biology, Methods in Molecular Biology 1021: 13–35. 23715978 10.1007/978-1-62703-450-0_2

[15] Saadatpour A, Albert R (2013) Boolean modeling of biological networks: A methodology tutorial. Methods 62: 3–12. 10.1016/j.ymeth.2012.10.012 23142247

[16] Kauffman S (1969) Homeostasis and differentiation in random genetic control networks. Nature 224: 177–178. 10.1038/224177a0 5343519

[17] Orth JD, Fleming RMT, Palsson BØ (2010) Reconstruction and use of microbial metabolic networks: the core escherichia coli metabolic model as an educational guide. EcoSal Plus. 10.1128/ecosalplus.10.2.1 26443778

[18] Wang RS, Saadatpour A, Albert R (2012) Boolean modeling in systems biology: an overview of methodology and applications. Physical Biology 9(5): 055001 10.1088/1478-3975/9/5/055001 23011283

[19] Fukuda K, Prodon A (1996) Double description method revisited. Combinatorics and Computer Science 1120: 91–111.

[20] Larhlimi A, David L, Selbig J, Bockmayr A (2012) F2C2: a fast tool for the computation of flux coupling in genome-scale metabolic networks. BMC Bioinformatics 13: 57 10.1186/1471-2105-13-57 22524245 PMC3515416

[21] Zolotykh NY (2012) New modification of the double describtion method for contstructing the skeleton of a polyhedral cone. Computational Mathematics and Mathematical Physics 52: 146–156. 10.1134/S0965542512010162

[22] Jensen PA, Lutz KA, Papin JA (2011) TIGER: Toolbox for integrating genome-scale metabolic models, expression data, and transcriptional regulatory networks. BMC Systems Biology 5: 147 10.1186/1752-0509-5-147 21943338 PMC3224351

[23] Jungreuthmayer C, Ruckerbauer DE, Zanghellini J (2013) regEfmtool: speeding up elementary flux mode calculation using transcriptional regulatory rules in the form of a three-state logic. Biosystems 113(1): 37–39. 10.1016/j.biosystems.2013.04.002 23664840

[24] University of California, San Diego, Systems Biology Research Group (2012) The Core *E. Coli* Model. http://gcrg.ucsd.edu/Downloads/EcoliCore.

[25] Jol SJ, Kümmel A, Terzer M, Stelling J, Matthias H (2012) System-level insights into yeast metabolism by thermodynamics analysis of elementary flux modes. PLoS Comput Biol 8(3): 1–9. 22416224 10.1371/journal.pcbi.1002415 PMC3296127

[26] van den Berg MA, de Jong-Gubbels P, Steensma HY (1998) Transient mRNA responses in chemostat cultures as a method of defining putative regulatory elements: Application to genes involved in *saccaromyces cerevisiae* acetyl-coeenzyme a metabolism. Yeast 14: 1089–1104. 10.1002/(SICI)1097-0061(19980915)14:12<1089::AID-YEA312>3.0.CO;2-K 9778795

[27] Terzer M (2009) Large scale methods to enumerate extreme rays and elementary modes. Ph.D. thesis, ETH Zurich.

[28] Palmieri L, Lasorsa FM, Vozza A, Agrimi G, Fiermonte G, Runswick MJ, et al (2000) Identification and functions of new transporters in yeast mitochondria. Biochimica et Biophysica Acta (BBA)—Bioenergetics 1459: 363–369. 10.1016/S0005-2728(00)00173-0 11004452

[29] Van Den Berg MA, Steensma HY (1995) ACS2, a saccharomyces cerevisiae gene encoding acetylcoenzyme a synthetase, essential for growth on glucose. European Journal of Biochemistry 231: 704–713. 10.1111/j.1432-1033.1995.tb20751.x 7649171

[30] Påhlman AK, Granath K, Ansell R, Hohmann S, Adler L (2001) The yeast glycerol 3-phosphatases gpp1p and gpp2p are required for glycerol biosynthesis and differentially involved in the cellular responses to osmotic, anaerobic, and oxidative stress. Journal of Biological Chemistry 276: 3555–3563. 10.1074/jbc.M007164200 11058591

[31] Mendoza L, Thieffry D, Alvarez-Buylla ER (1999) Genetic control of ower morphogenesis in *arabidopsis thaliana*: a logical analysis. Bioinformatics 15: 593–606. 10.1093/bioinformatics/15.7.593 10487867

[32] Albert R, Othmer HG (2003) The topology of the regulatory interactions predicts the expression pattern of the segment polarity genes in *drosophilia melanogaster* . Journal of TheoreticalBiology 223: 1–18. 10.1016/s0022-5193(03)00035-3 PMC6388622 12782112

[33] Herrgård MH, Lee BS, Portnoy V, Palsson BØ (2006) Integrated analysis of regulatory and metabolic network reveals novel regulator mechanismn *saccaromyces cerevisiae* . Genome Research 16(5): 627–635. 10.1101/gr.4083206 16606697 PMC1457053

[34] Christensen TS, Soberano de Oliveira AP, Nielsen J (2009) Reconstruction and logical modeling of glucose repression signaling pathways in *saccaromyces cerevisiae* . BMC Systems Biology 3:7: 1–15. 19144179 10.1186/1752-0509-3-7 PMC2661888

[35] Meister M, Saum S, Alber BE, Fuchs G (2005) L-malyl-coenzyme a/beta-methylmalyl-coenzyme a lyase is involved in acetate assimilation of the isocitrate lyase-negative bacterium rhodobacter capsulatus. Journal of Bacteriology 187: 1415–1425. 10.1128/JB.187.4.1415-1425.2005 15687206 PMC545638

[36] Han L, Reynolds KA (1997) A novel alternate anaplerotic pathway to the glyoxylate cycle in streptomycetes. Journal of Bacteriology 179: 5157–5164. 9260959 10.1128/jb.179.16.5157-5164.1997 PMC179375

[37] Claassen PAM, Zehnder AJB (1986) Isocitrate lyase activity in thiobacillus versutus grown anaerobically on acetate and nitrate. Journal of General Microbiology 132: 3179–3185.

[38] Thomas R, D’Ari R (1990) Biological Feedback. Boca Raton: CRC Press.

[39] Shlomi T, Eisenberg Y, Sharan R, Ruppin E (2007) A genome-scale computational study of the interplay between transcriptional regulation and metabolism. Molecular System Biology 3: 101. 10.1038/msb4100141 PMC1865583 17437026

[40] Kaleta C, de Figueiredo LF, Schuster S (2009) Can the whole be less than the sum of its parts? pathway analysis in genome-scale metabolic networks using elementary flux patterns. Genome Res 19: 1872–1873. 10.1101/gr.090639.108 19541909 PMC2765277

[41] Kaleta C, De Figueiredo LF, Behre J, Schuster S (2009) EFMEvolver: computing elementary flux modes in genome-scale metabolic networks In: Lecture Notes in Informatics (LNI) P-157—Proceedings of the German Conference on Bioinformatics. Bonn: Gesellschaft fr Informatik, pp. 179–190.

[42] Machado D, Soons Z, Patil KR, Ferreira EC, Rocha I (2012) Random sampling of elementary flux modes in large-scale metabolic networks. Bioinformatics 28:18: I515–I521. 10.1093/bioinformatics/bts401 22962475 PMC3436828

[43] Tabe-Bordbar S, Marashi SA (2013) Finding elementary flux modes in metabolic networks based on flux balance analysis and flux coupling analysis: application to the analysis of *Escherichia coli* metabolism. Biotechnol Lett 35:12: 2039–2044. 10.1007/s10529-013-1328-x 24078125

[44] Pey J, Planes FJ (2014) Direct calculation of elementary flux modes satisfying several biological constraints in genome-scale metabolic networks. Systems Biology in press: 1–7. 10.1093/bioinformatics/btu193 24728852

[45] Schwartz JM, Kanehisa M (2006) Quantitative elementary mode analysis of metabolic pathways: the example of yeast glycolysis. BMC Bioinformatics 7: 1–20. 10.1186/1471-2105-7-1 16584566 PMC1508158

[46] Jungreuthmayer C, Zanghellini J (2012) Designing optimal cell factories: integer programming couples elementary mode analysis with regulation. BMC Systems Biology 6: 103 10.1186/1752-0509-6-103 22898474 PMC3560272

[47] Hädicke O, Klamt S (2011) Computing complex metabolic intervention strategies using constrained minimal cut sets. Metabolic Engineering 13: 204–213. 10.1016/j.ymben.2010.12.004 21147248

[48] von Kamp A, Klamt S (2014) Enumeration of smallest intervention strategies in genome-scale metabolic networks. PLoS Comput Biol 10: e1003378 10.1371/journal.pcbi.1003378 24391481 PMC3879096

[49] Jungreuthmayer C, Beurton-Aimar M, Zanghellini J (2013) Fast computation of minimal cut sets in metabolic networks with a berge algorithm that utilizes binary bit pattern trees. IEEE/ACM Transactions on Computational Biology and Bioinformatics 10: 1329–33. 10.1109/TCBB.2013.116 24062540

[50] Jungreuthmayer C, Nair G, Klamt S, Zanghellini J (2013) Comparison and improvement of algorithms for computing minimal cut sets. BMC Bioinformatics 14: 318 10.1186/1471-2105-14-318 24191903 PMC3882775

[51] Covert MW, Knight EM, Reed JL, Herrgard MJ, Palsson BO (2004) Integrating high-throughput and computational data elucidates bacterial networks. Nature 429: 92–96. 10.1038/nature02456 15129285

[52] Chandrasekaran S, Price ND (2010) Probabilistic integrative modeling of genome-scale metabolic and regulatory networks in escherichia coli and mycobacterium tuberculosis. Proceedings of the National Academy of Sciences. 10.1073/pnas.1005139107 PMC2955152 20876091

