# Supplementary figures and images for: Avoiding the Enumeration of Infeasible Elementary Flux Modes by Including Transcriptional Regulatory Rules in the Enumeration Process Saves Computational Costs
Source: PLoS One. 2015 Jun 19;10(6):e0129840. doi: 10.1371/journal.pone.0129840 (PMC4475075; doi:10.1371/journal.pone.0129840)

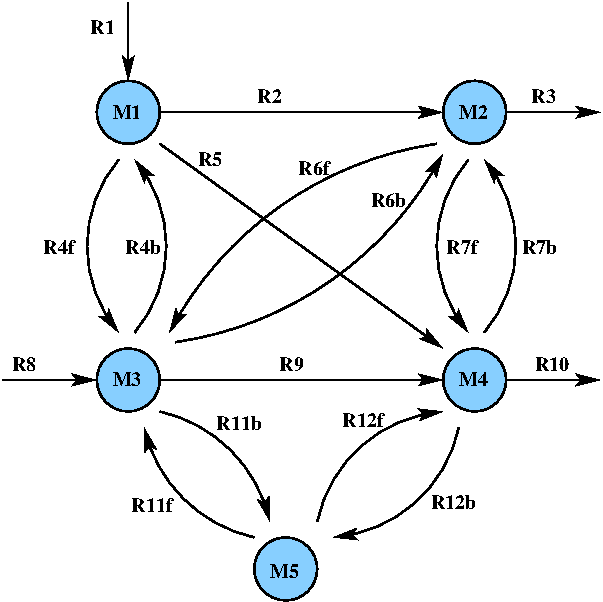

Supplement: S2 Fig — The network consists of five metabolites and twelve reactions of which five are reversible and, hence, split in non-reversible forward and backward reactions. The stoichiometric matrix of the example is shown in S6 Table. The network has 30 elementary flux modes if no gene rules are used to restrict the solution space (see S8 Table). If the rule R1 = (!fR5) is applied, the network contains only eleven modes. (TIFF) [file pone.0129840.s002.tiff]

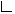

Supplement: S4 File — (GZ) [file pone.0129840.s014.gz › 20120810_regEfmtool_2.0/javadoc/resources/inherit.gif]
