# Supplementary material for: Avoiding the Enumeration of Infeasible Elementary Flux Modes by Including Transcriptional Regulatory Rules in the Enumeration Process Saves Computational Costs
Source: PLoS One. 2015 Jun 19;10(6):e0129840. doi: 10.1371/journal.pone.0129840 (PMC4475075; doi:10.1371/journal.pone.0129840)
Supplement: S1 Table — External metabolites which are irrelevant for the calculation of the elementary flux modes are omitted. (PDF) [file pone.0129840.s003.pdf]

Table S1: Stoichiometric matrix, S, of the example network. External metabolites which are irrelevant for the calculation of the elementary flux modes are omitted.

	R1	R2	R3	R4	R5	R6	R7r	R8	R9	R10	R11
A	1.0	-0.5	0.0	0.0	0.0	0.0	-1.0	0.0	0.0	-1.0	0.0
C	0.0	1.0	1.0	0.0	0.0	0.0	1.0	-1.0	0.0	0.0	-1.0
D	0.0	0.0	0.0	0.0	0.0	0.0	0.0	0.0	-2.0	1.0	1.0
P	0.0	0.0	0.0	-1.0	0.0	0.0	0.0	1.0	1.0	0.0	0.0
Q	0.0	0.0	0.0	0.0	-1.0	0.0	0.0	0.0	0.0	0.0	1.0
S	0.0	0.0	0.0	0.0	0.0	-1.0	0.0	0.0	0.0	1.0	0.0
