# Supplementary material for: Avoiding the Enumeration of Infeasible Elementary Flux Modes by Including Transcriptional Regulatory Rules in the Enumeration Process Saves Computational Costs
Source: PLoS One. 2015 Jun 19;10(6):e0129840. doi: 10.1371/journal.pone.0129840 (PMC4475075; doi:10.1371/journal.pone.0129840)
Supplement: S2 Table — (PDF) [file pone.0129840.s004.pdf]

Table S2: Extended stoichiometric matrix, S_{ext}, of the example network shown in Fig. 1 after splitting the reversible reaction R7r into the two irreversible reactions R7f and R7b.

	R1	R2	R3	R4	R5	R6	R7f	R7b	R8	R9	R10	R11
A	1.0	-0.5	0.0	0.0	0.0	0.0	-1.0	1.0	0.0	0.0	-1.0	0.0
C	0.0	1.0	1.0	0.0	0.0	0.0	1.0	-1.0	-1.0	0.0	0.0	-1.0
D	0.0	0.0	0.0	0.0	0.0	0.0	0.0	0.0	0.0	-2.0	1.0	1.0
P	0.0	0.0	0.0	-1.0	0.0	0.0	0.0	0.0	1.0	1.0	0.0	0.0
Q	0.0	0.0	0.0	0.0	-1.0	0.0	0.0	0.0	0.0	0.0	0.0	1.0
S	0.0	0.0	0.0	0.0	0.0	-1.0	0.0	0.0	0.0	0.0	1.0	0.0
