# Supplementary material for: Avoiding the Enumeration of Infeasible Elementary Flux Modes by Including Transcriptional Regulatory Rules in the Enumeration Process Saves Computational Costs
Source: PLoS One. 2015 Jun 19;10(6):e0129840. doi: 10.1371/journal.pone.0129840 (PMC4475075; doi:10.1371/journal.pone.0129840)
Supplement: S3 Table — 1 means that the reaction carries a flux and 0 means the reaction carries no flux. Note that the futile two-cycle of the reversible reaction R7r has already been removed and the forward and backward irreversible reactions (R7f and Rfb) have been combined to the reversible reaction R7r by a bitwise OR operation. (PDF) [file pone.0129840.s005.pdf]

Table S3: Binary representation of all elementary flux modes of the example network shown in Fig. 1. 1 means that the reaction carries a flux and 0 means the reaction carries no flux. Note that the futile two-cycle of the reversible reaction *R7r* has already been removed and the forward and backward irreversible reactions (*R7f* and *Rfb*) have been combined to the reversible reaction *R7r* by a bitwise OR operation.

	R1	R2	R3	R4	R5	R6	R7r	R8	R9	R10	R11
EFM01	0	1	0	1	0	0	1	1	0	0	0
EFM02	0	1	0	1	1	0	1	0	1	0	1
EFM03	0	1	0	1	0	1	1	0	1	1	0
EFM04	0	0	1	1	0	1	1	0	0	1	0
EFM05	1	1	0	1	0	0	0	1	0	0	0
EFM06	1	1	0	1	1	0	0	0	1	0	1
EFM07	1	0	0	1	0	1	0	0	1	1	0
EFM08	1	0	0	1	1	0	1	0	1	0	1
EFM09	1	0	0	1	0	0	1	1	0	0	0
EFM10	0	0	1	1	1	0	0	0	1	0	1
EFM11	0	0	1	1	0	0	0	1	0	0	0
