# Supplementary material for: Avoiding the Enumeration of Infeasible Elementary Flux Modes by Including Transcriptional Regulatory Rules in the Enumeration Process Saves Computational Costs
Source: PLoS One. 2015 Jun 19;10(6):e0129840. doi: 10.1371/journal.pone.0129840 (PMC4475075; doi:10.1371/journal.pone.0129840)
Supplement: S4 Table — The values of the reactions are shown in columns mig1, mth1, gln, rgt1. The characters ‘⇒’ and ‘⇔’ denote the explicit and implicit formulation as defined by Jensen et al. Feasible combinations of reactions are highlighted in color. For the undefined state of the three-state logic the character ‘u’ is used. (PDF) [file pone.0129840.s006.pdf]

Table S4: Comparison of four methods of genetic rule formulations: (i) explicit by Jensen et al., (ii) implicit by Jensen et al., (iii) three state logic - implicit functionality, and (iv) three state logic - direct implementation. The values of the reactions are shown in columns mig1, mth1, gln, rgt1. The characters '⇒' and '⇔' denote the explicit and implicit formulation as defined by Jensen et al. Feasible combinations of reactions are highlighted in color. For the *undefined* state of the three-state logic the character 'u' is used.

truth table				explicit - Jensen et al.		implicit - Jensen et al.			three state - implicit functionality			three state - direct implementation	
mig1	mth1	gln	rgt1	NOT mig1 ⇔mth1	mth1 AND (NOT gln) ⇔rgt1	mig1 ⇒NOT mth1	mth1 ⇒rgt1	gln ⇒NOT rgt1	mth1 = NOT 1-mig1	rgt1 = 1-mth1	rgt1 = NOT 1-gln	mth1= NOT 1-mig1	rgt1 = 1-mth1 AND (NOT 1-gln)
0	0	0	0	1 ≠ 0	0 & 1 = 0 ≡ 0: feasible	**0 ⇒1: feasible**	**0 ⇒0: feasible**	**0 ⇒1: feasible**	**0 ≡ u: feasible**	**0 ≡ u: feasible**	**0 ≡ u: feasible**	**0 ≡ u: feasible**	**0 ≡ u & u = u: feasible**
0	0	0	1	1 ≠ 0	0 & 1 = 0 ≠ 1	**0 ⇒1: feasible**	**0 ⇒1: feasible**	**0 ⇒0: feasible**	**0 ≡ u: feasible**	**1 ≡ u: feasible**	**1 ≡ u: feasible**	**0 ≡ u: feasible**	**1 ≡ u & u = u: feasible**
0	0	1	0	1 ≠ 0	0 & 0 = 0 ≡ 0: feasible	**0 ⇒1: feasible**	**0 ⇒0: feasible**	**1 ⇒1: feasible**	**0 ≡ u: feasible**	**0 ≡ u: feasible**	**0 ≡ 0: feasible**	**0 ≡ u: feasible**	**0 ≡ u & 0 = 0: feasible**
0	0	1	1	1 ≠ 0	0 & 0 = 0 ≠ 1	0 ⇒1: feasible	0 ⇒1: feasible	1 ⇒0	0 ≡ u: feasible	1 ≡ u: feasible	1 ≠ 0	0 ≡ u: feasible	1 ≡ u & 0 = 0
0	1	0	0	1 ≡ 1: feasible	1 & 1 = 1 ≠ 0	0 ⇒0: feasible	1 ⇒0	0 ⇒1: feasible	1 ≡ u: feasible	0 ≠ 1	0 ≡ u: feasible	**1 ≡ u: feasible**	**0 ≡ 1 & u = u: feasible**
0	1	0	1	**1 ≡ 1: feasible**	**1 & 1 = 1 ≡ 1: feasible**	**0 ⇒0: feasible**	**1 ⇒1: feasible**	**0 ⇒0: feasible**	**1 ≡ u: feasible**	**1 ≡ 1: feasible**	**1 ≡ u: feasible**	**1 ≡ u: feasible**	**1 ≡ 1 & u = u: feasible**
0	1	1	0	**1 ≡ 1: feasible**	**1 & 0 = 0 ≡ 0: feasible**	0 ⇒0: feasible	1 ⇒0	1 ⇒1: feasible	1 ≡ u: feasible	0 ≠ 1	0 ≡ 0: feasible	**1 ≡ u: feasible**	**0 ≡ 1 & 0 = 0: feasible**
0	1	1	1	1 ≡ 1: feasible	1 & 0 = 0 ≠ 1	0 ⇒0: feasible	1 ⇒1: feasible	1 ⇒0	1 ≡ u: feasible	1 ≡ 1: feasible	1 ≠ 0	1 ≡ u: feasible	1 ≠ 1 & 0 = 0
1	0	0	0	**0 ≡ 0: feasible**	**0 & 1 = 0 ≡ 0: feasible**	**1 ⇒1: feasible**	**0 ⇒0: feasible**	**0 ⇒1: feasible**	**0 ≡ 0: feasible**	**0 ≡ u: feasible**	**0 ≡ u: feasible**	**0 ≡ 0: feasible**	**0 ≡ u & u = u: feasible**
1	0	0	1	0 ≡ 0: feasible	0 & 1 = 0 ≠ 1	**1 ⇒1: feasible**	**0 ⇒1: feasible**	**0 ⇒0: feasible**	**0 ≡ 0: feasible**	**1 ≡ u: feasible**	**1 ≡ u: feasible**	**0 ≡ 0: feasible**	**1 ≡ u & u = u: feasible**
1	0	1	0	**0 ≡ 0: feasible**	**0 & 0 = 0 ≡ 0: feasible**	**1 ⇒1: feasible**	**0 ⇒0: feasible**	**1 ⇒1: feasible**	**0 ≡ 0: feasible**	**0 ≡ u: feasible**	**0 ≡ 0: feasible**	**0 ≡ 0: feasible**	**0 ≡ u & 0 = 0: feasible**
1	0	1	1	0 ≡ 0: feasible	0 & 0 = 0 ≠ 1	1 ⇒1: feasible	0 ⇒1: feasible	1 ⇒0	0 ≡ 0: feasible	1 ≡ u: feasible	1 ≠ 0	0 ≡ 0: feasible	1 ≠ u & 0 = 0
1	1	0	0	0 ≠ 1	1 & 1 = 1 ≠ 0	1 ⇒0	1 ⇒0	0 ⇒1: feasible	1 ≠ 0	0 ≠ 1	0 ≡ u: feasible	1 ≠ 0	0 ≡ 1 & u = u: feasible
1	1	0	1	0 ≠ 1	1 & 1 = 1 ≡ 1: feasible	1 ⇒0	1 ⇒1: feasible	0 ⇒0: feasible	1 ≠ 0	1 ≡ 1: feasible	1 ≡ u: feasible	1 ≠ 0	1 ≡ 1 & u = u: feasible
1	1	1	0	0 ≠ 1	1 & 0 = 0 ≡ 0: feasible	1 ⇒0	1 ⇒0	1 ⇒1: feasible	1 ≠ 0	0 ≠ 1	0 ≡ 0: feasible	1 ≠ 0	0 ≡ 1 & 0 = 0: feasible
1	1	1	1	0 ≠ 1	1 & 0 = 0 ≠ 1	1 ⇒0	1 ⇒1: feasible	1 ⇒0	1 ≠ 0	1 ≡ 1: feasible	1 ≠ 0	1 ≠ 0	1 ≠ 1 & 0 = 0

The table shows a comparison of four regulatory rule formulations. *explicit - Jensen et al.* and *implicit - Jensen et al.* show formulation concepts that have been reported by Jensen et al. to illustrate the problem of over-constraining rule sets. The implicit formulation allows a larger number of feasible combinations of input reactions compared to the explicit formulation. The explicit formulation by Jensen et al. is identical to our method when all reactions are set to full-activity. The formulation *three state logic - implicit functionality* uses our formulation to implement the implicit approach by Jensen et al. If our approach is directly implemented using the three state logic an even more relaxed rule set can be defined. S4 Table shows that the approach of using a three-state logic and reaction activities (0-active, 1-active, and full-active) offers a similar flexibility, as the implicit formulation reported by Jensen et al. In S4 Table the notation '1-*reaction_name*' indicates that a reaction is 1-active. A rule that evaluates to *undefined* is treated as feasible, as the rule cannot be used to eliminate the mode based on an *undefined* state.
