# Supplementary material for: Avoiding the Enumeration of Infeasible Elementary Flux Modes by Including Transcriptional Regulatory Rules in the Enumeration Process Saves Computational Costs
Source: PLoS One. 2015 Jun 19;10(6):e0129840. doi: 10.1371/journal.pone.0129840 (PMC4475075; doi:10.1371/journal.pone.0129840)
Supplement: S5 Table — Text sections in italic style indicate our modifications compared to the binary method reported by Gagneur and Klamt [5]. (PDF) [file pone.0129840.s007.pdf]

Table S5: Pseudocode for the *regEFMtool*. Text sections in *italic* style indicate our modifications compared to the binary method reported by Gagneur and Klamt [5].

Pre-processing phase

Read input files (stoichiometric matrix, reversibility, ...)
Split each reversible reaction into two irreversible reactions
Read file containing gene regulatory information
Parse gene regulation file
LOOP: Iterate through all provided rules
    Create reverse polish notation (RPN) stack for rule
    Add RPN stack to post-processing rule container
    IF rule qualifies for iteration phase THEN
        Create bit pattern for rule
        Add bit pattern to iteration phase rule container
    END IF
END OF LOOP
Compute kernel K of stoichiometric matrix
Use kernel K to initialize mode matrix R
Sort mode matrix R (put rows with only positive elements to top)
Convert all rows with only positive elements to binary format

Iteration phase

LOOP1: Iterate through all numerical rows of mode matrix R
    LOOP2: Iterate through all adjacent positive-negative pairs
        Create new mode by combining parent modes
        LOOP3: Iterate through all iteration phase rules
            IF mode does not obey rule THEN
                Eliminate mode
                LAST LOOP 3
            END IF
        END OF LOOP3
    END OF LOOP2
    Remove all modes with negative values at current row
    Convert current row to binary format
END OF LOOP1

Post-processing phase

LOOP1: Iterate through all modes
    LOOP2: Iterate through all post-processing rules
        IF mode does not obey rule THEN
            Remove mode
            LAST LOOP2
        END IF
    END OF LOOP2
END OF LOOP1
Remove futile 2-cycle modes
Convert binary modes to numerical form
Write result file
