# Supplementary material for: Avoiding the Enumeration of Infeasible Elementary Flux Modes by Including Transcriptional Regulatory Rules in the Enumeration Process Saves Computational Costs
Source: PLoS One. 2015 Jun 19;10(6):e0129840. doi: 10.1371/journal.pone.0129840 (PMC4475075; doi:10.1371/journal.pone.0129840)
Supplement: S6 Table — (PDF) [file pone.0129840.s008.pdf]

Table S6: Extended stoichiometric matrix S_{ext} of the example network shown in S2 Fig.

	R1	R2	R3	R4f	R4b	R5	R6f	R6b	R7f	R7b	R8	R9	R10	R11f	R11b	R12f	R12b
M1	1	-1	0	-1	1	-1	0	0	0	0	0	0	0	0	0	0	0
M2	0	1	-1	0	0	0	-1	1	-1	1	0	0	0	0	0	0	0
M3	0	0	0	1	-1	0	1	-1	0	0	1	-1	0	1	-1	0	0
M4	0	0	0	0	0	1	0	0	0	0	0	1	-1	0	0	1	-1
M5	0	0	0	0	0	0	0	0	0	0	0	0	0	-1	1	-1	1
