# Supplementary material for: Avoiding the Enumeration of Infeasible Elementary Flux Modes by Including Transcriptional Regulatory Rules in the Enumeration Process Saves Computational Costs
Source: PLoS One. 2015 Jun 19;10(6):e0129840. doi: 10.1371/journal.pone.0129840 (PMC4475075; doi:10.1371/journal.pone.0129840)
Supplement: S7 Table — (PDF) [file pone.0129840.s009.pdf]

Table S7: Mode matrix R of a simple example network after sorting the reactions and before executing the iteration phase.

Reaction name	intermediate modes												Number of positive values	Number of negative values	Number of adjacency candidates
R3	t	f	f	f	f	f	f	f	f	f	f	f	1	0	0
R6f	f	t	f	f	f	f	f	f	f	f	f	f	1	0	0
R6b	f	f	t	f	f	f	f	f	f	f	f	f	1	0	0
R7f	f	f	f	t	f	f	f	f	f	f	f	f	1	0	0
R7b	f	f	f	f	t	f	f	f	f	f	f	f	1	0	0
R8	f	f	f	f	f	t	f	f	f	f	f	f	1	0	0
R9	f	f	f	f	f	f	t	f	f	f	f	f	1	0	0
R10	f	f	f	f	f	f	f	t	f	f	f	f	1	0	0
R4b	f	f	f	f	f	f	f	f	t	f	f	f	1	0	0
R11b	f	f	f	f	f	f	f	f	f	t	f	f	1	0	0
R12f	f	f	f	f	f	f	f	f	f	f	t	f	1	0	0
R12b	f	f	f	f	f	f	f	f	f	f	f	t	1	0	0
R11f	0.0	0.0	0.0	0.0	0.0	0.0	0.0	0.0	0.0	1.0	-1.0	1.0	2	1	2
R5	0.0	0.0	0.0	0.0	0.0	0.0	-1.0	1.0	0.0	0.0	-1.0	1.0	2	2	4
R1	1.0	0.0	0.0	1.0	-1.0	-1.0	0.0	1.0	0.0	0.0	0.0	0.0	3	2	6
R2	1.0	1.0	-1.0	1.0	-1.0	0.0	0.0	0.0	0.0	0.0	0.0	0.0	3	2	6
R4f	0.0	-1.0	1.0	0.0	0.0	-1.0	1.0	0.0	1.0	0.0	1.0	-1.0	4	3	12
