# Supplementary material for: Avoiding the Enumeration of Infeasible Elementary Flux Modes by Including Transcriptional Regulatory Rules in the Enumeration Process Saves Computational Costs
Source: PLoS One. 2015 Jun 19;10(6):e0129840. doi: 10.1371/journal.pone.0129840 (PMC4475075; doi:10.1371/journal.pone.0129840)
Supplement: S8 Table — (PDF) [file pone.0129840.s010.pdf]

Table S8: The complete set of elementary modes of the example network shown in S2 Fig. if no gene rules are used to restrict the solution space.

R1	R2	R3	R4f	R4b	R5	R6f	R6b	R7f	R7b	R8	R9	R10	R11f	R11b	R12f	R12b
0.0	0.0	0.0	0.0	1.0	1.0	1.0	0.0	0.0	1.0	0.0	0.0	1.0	0.0	0.0	0.0	0.0
1.0	0.0	0.0	0.0	0.0	1.0	0.0	1.0	1.0	0.0	0.0	0.0	0.0	1.0	0.0	0.0	1.0
1.0	0.0	1.0	0.0	0.0	1.0	0.0	1.0	0.0	0.0	0.0	0.0	0.0	1.0	0.0	0.0	1.0
0.0	0.0	0.0	0.0	1.0	1.0	0.0	0.0	0.0	0.0	0.0	0.0	0.0	1.0	0.0	0.0	1.0
1.0	1.0	0.0	0.0	0.0	0.0	1.0	0.0	0.0	0.0	0.0	0.0	1.0	0.0	1.0	1.0	0.0
1.0	1.0	0.0	0.0	0.0	0.0	1.0	0.0	0.0	0.0	0.0	1.0	1.0	0.0	0.0	0.0	0.0
0.0	1.0	0.0	0.0	1.0	0.0	0.0	0.0	1.0	0.0	1.0	0.0	0.0	0.0	0.0	0.0	0.0
0.0	1.0	1.0	0.0	1.0	0.0	0.0	0.0	0.0	0.0	1.0	0.0	0.0	0.0	0.0	0.0	0.0
0.0	1.0	0.0	0.0	1.0	0.0	1.0	0.0	0.0	0.0	0.0	0.0	0.0	0.0	0.0	0.0	0.0
0.0	0.0	0.0	0.0	1.0	1.0	0.0	0.0	0.0	0.0	1.0	0.0	1.0	0.0	0.0	0.0	0.0
1.0	0.0	0.0	1.0	0.0	0.0	0.0	0.0	0.0	0.0	0.0	1.0	1.0	0.0	0.0	0.0	0.0
1.0	0.0	0.0	1.0	0.0	0.0	0.0	0.0	0.0	0.0	0.0	0.0	1.0	0.0	1.0	1.0	0.0
0.0	0.0	0.0	1.0	1.0	0.0	0.0	0.0	0.0	0.0	0.0	0.0	0.0	0.0	0.0	0.0	0.0
1.0	0.0	1.0	1.0	0.0	0.0	0.0	1.0	0.0	0.0	0.0	0.0	0.0	0.0	0.0	0.0	0.0
1.0	0.0	0.0	1.0	0.0	0.0	0.0	1.0	1.0	0.0	0.0	0.0	0.0	0.0	0.0	0.0	0.0
1.0	1.0	1.0	0.0	0.0	0.0	0.0	0.0	0.0	0.0	0.0	0.0	0.0	0.0	0.0	0.0	0.0
1.0	1.0	0.0	0.0	0.0	0.0	0.0	0.0	1.0	0.0	0.0	0.0	0.0	0.0	0.0	0.0	0.0
1.0	0.0	0.0	0.0	0.0	1.0	0.0	0.0	0.0	0.0	0.0	0.0	1.0	0.0	0.0	0.0	0.0
0.0	0.0	0.0	0.0	0.0	0.0	0.0	0.0	0.0	0.0	0.0	0.0	0.0	1.0	1.0	0.0	0.0
0.0	0.0	0.0	0.0	0.0	0.0	0.0	0.0	0.0	0.0	0.0	0.0	0.0	0.0	0.0	1.0	1.0
0.0	0.0	0.0	0.0	0.0	0.0	0.0	0.0	0.0	0.0	0.0	1.0	0.0	1.0	0.0	0.0	1.0
0.0	0.0	0.0	0.0	0.0	0.0	0.0	0.0	1.0	1.0	0.0	0.0	0.0	0.0	0.0	0.0	0.0
0.0	0.0	1.0	0.0	0.0	0.0	0.0	0.0	0.0	1.0	0.0	0.0	0.0	0.0	0.0	0.0	0.0
0.0	0.0	0.0	0.0	0.0	0.0	0.0	0.0	0.0	0.0	1.0	0.0	1.0	0.0	1.0	1.0	0.0
0.0	0.0	0.0	0.0	0.0	0.0	1.0	0.0	0.0	1.0	0.0	0.0	1.0	0.0	1.0	1.0	0.0
0.0	0.0	0.0	0.0	0.0	0.0	1.0	0.0	0.0	1.0	0.0	1.0	1.0	0.0	0.0	0.0	0.0
0.0	0.0	1.0	0.0	0.0	0.0	0.0	1.0	0.0	0.0	1.0	0.0	0.0	0.0	0.0	0.0	0.0
0.0	0.0	0.0	0.0	0.0	0.0	0.0	1.0	1.0	0.0	1.0	0.0	0.0	0.0	0.0	0.0	0.0
0.0	0.0	0.0	0.0	0.0	0.0	1.0	1.0	0.0	0.0	0.0	0.0	0.0	0.0	0.0	0.0	0.0
0.0	0.0	0.0	0.0	0.0	0.0	0.0	0.0	0.0	0.0	1.0	1.0	1.0	0.0	0.0	0.0	0.0
